# Revealing the Hidden Polysulfides in Solid-State Na–S Batteries: How Pressure and Electrical Transport Control Kinetic Pathways

**DOI:** 10.1021/jacs.5c00465

**Published:** 2025-06-23

**Authors:** Hung Quoc Nguyen, Mikael Dahl Kanedal, Juraj Todt, Feng Jin, Quyen Do, Dora Zalka, Alexey Maximenko, Dragos Stoian, Norbert Schell, Wouter van Beek, Harald Fitzek, Johannes Rattenberger, Valerie Siller, Steven T. Boles, Mario El Kazzi, Jozef Keckes, Daniel Rettenwander

**Affiliations:** † Department of Materials Science and Engineering, 8018 NTNU Norwegian University of Science and Technology, Trondheim 7034, Norway; ‡ Chair of Materials Physics, 27268 Montanuniversität Leoben and Erich Schmid Institute for Materials Science, Austrian Academy of Sciences, Leoben 8700, Austria; § Department of Energy and Process Engineering, Norwegian University of Science and Technology, Trondheim 7491, Norway; ∥ National Synchrotron Radiation Centre SOLARIS, 37799 Jagiellonian University, Czerwone Maki 98, Kraków 30-392, Poland; ⊥ Swiss-Norwegian Beamlines, 55553 European Synchrotron Radiation Facility, 71 Ave. des Martyrs, Grenoble 38000, France; # 28338 Helmholtz-Zentrum Hereon, Max-Planck-Straße 1, Geesthacht 21502, Germany; ¶ Graz Centre for Electron Microscopy (ZFE), Steyrergasse 17, Graz 8010, Austria; ∇ PSI Center for Energy and Environmental Sciences, Villigen PSI 5232, Switzerland; ○ Christian Doppler Laboratory for Solid State Batteries, Norwegian University of Science and Technology, Trondheim 7034, Norway; ⧫ AIT Austrian Institute of Technology GmbH, Center for Transport Technologies, Battery Technologies, Vienna 1210, Austria

## Abstract

Room temperature operation of Na–S batteries with liquid electrolytes is plagued by fundamental challenges stemming from polysulfide solubility and their shuttle effects. Inorganic solid electrolytes offer a promising solution by acting as barriers to polysulfide migration, mitigating capacity loss. While the sequential formation of cycling products in molten-electrode and liquid electrolytes-based Na–S batteries generally aligns with the expectations from the Na–S phase diagram, their presence, stability, and transitory behavior in systems with inorganic solid electrolytes at room temperature, remain poorly understood. To address this, we employed operando scanning microbeam X-ray diffraction, operando X-ray photoelectron spectroscopy and ex-situ X-ray absorption spectroscopy to investigate the sulfur conversion mechanisms in Na–S cells with Na_3_PS_4_ and Na_4_(B_10_H_10_)­(B_12_H_12_) electrolytes. Our findings reveal the formation of crystalline and amorphous polysulfides, including those predicted by the Na–S phase diagram (e.g., Na_2_S_5_, Na_2_S_4_, Na_2_S_2_, Na_2_S), high-order polysulfides observed in liquid-electrolyte systems (e.g., Na_2_S_*x*_, where *x* = 6–8), and phases like Na_2_S_3_ typically stable only under high-temperature or high-pressure conditions. We demonstrate that these transitions are governed by diffusion-limited kinetics and localized stress concentrations, emphasizing the critical role of pressure, which serves as both a thermodynamic variable, as well as a design parameter, for optimizing solid-state Na–S battery performance necessary for pushing these cells closer to the commercial frontier.

## Graphical abstract

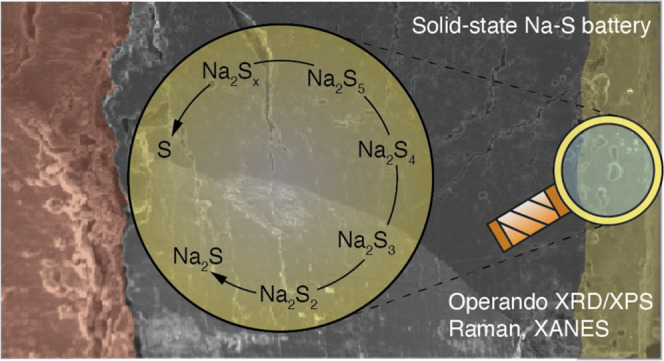

## 1 Introduction

Na–S batteries, despite their relatively limited commercial deployment in recent years, have a substantial history in mobile energy storage, primarily due to their high energy density, abundance of sodium and sulfur, and potential for cost-effectiveness.^1^ Before the rise of lithium-based chemistries and sustainability-driven policies, Na–S cells held promise across sectors, notably in the global automotive industry. This promise was bolstered by the low melting points of Na and S and the discovery of rapid Na-ion transport in β’’-alumina, which catalyzed interest in liquid-electrode systems.^2^ However, recent advances in room-temperature-compatible aprotic electrolytes have revived the interest in Na–S batteries that function at ambient temperatures.^3^

Recent Na–S systems with heavily ‘cocktail’-optimized liquid electrolytes have exhibited impressive performance, showing an initial capacity of 1635 mAh/g (sulfur weight basis) at 0.1C, with 56.7% capacity retention over 200 cycles and high Coulombic efficiency.^4^ However, even these advancements fall short of the high-performance criteria required for large-scale energy storage and mobility applications, particularly given the 10+ year lifespans now standard for conventional Li-ion batteries.^5^ This raises the question of whether electrolyte engineering alone can overcome the fundamental challenges inherent in sulfur-cathode cells, such as (i) polysulfide dissolution and shuttle effects and (ii) significant volume changes during redox cycling, both of which contribute to chemo-mechanical degradation at the cathode–electrolyte interface. The analogous Li–S battery system has faced similarly challenges, prompting growing research efforts to optimize the cathode structure and interface for enhanced compatibility with liquid electrolytes.^6–8^

An alternative approach involves integrating inorganic solid electrolytes, which could intrinsically address polysulfide sodiation reversibility.^9^ The particulate morphology of solid-state materials, combined with carbon-black mixing, constrains sulfur hosts within a stable position, removing some of the risks associated with dissolution. This stability contrasts with liquid systems, where electrolyte fluid mechanics makes dissolution catastrophic. In liquid cells, a complex ″solid–liquid–solid″ transformation occurs during cycling, involving long-chain (soluble) and short-chain (nonsoluble) polysulfides with characteristic discharge plateaus.^10^ More recently, some researchers have turned to novel electrolyte engineering approaches to alter the electrochemical pathways that are responsible for the polysulfide-driven problems. For example, Qian et al. have reported that localized high-concentration electrolytes with novel salt-solvent formulations which allow lead to new cathode electrolyte interphases, and hence, evidence of “solid–solid” transformations enabled in situ.^11^ While overall this direction is still developing, it is clear that moving away from liquid-mediated sulfur conversion is critical to controlling the stability and materials and interfaces in this cell type.

In solid-state Na–S cells, however, the physical stability and variable stack pressure of solid components provide a different, albeit challenging, chemo-mechanical environment, which is similar to the Li–S cells.^12,13^ Mechanisms for transforming S to Na_2_S in solid systems are anticipated to resemble those in liquid and molten Na–S cells but with unique kinetic dynamics. Observations of solid–solid reactions in solid-state Na–S batteries hint at similar behaviors, though the existence of polysulfide intermediates remains poorly understood (see Figure 1a,b).^9^

**Figure 1:**
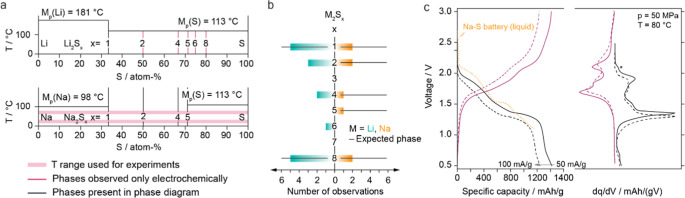
Phase behavior of M-S systems and comparison with experimental electrochemical measurements. (a) Zoomed-in phase diagrams of the Li–S (a)^47^ and Na–S (b)^48^ systems, showing the relevant temperature ranges, excluding regions containing liquid phases. The pink-shaded area represents the temperature range used in this study to investigate polysulfide formation. (b) Schematic depicting the theoretically possible polysulfides (M_2_S_*x*_, with M = Li, Na, and *x* ∈ {1, 2, ..., 8},^33^ and the frequency of observation of these polysulfides in M-S solid-state batteries with inorganic electrolytes.^14,21,35,36,49–51^ (c) Voltage profile and corresponding dq/dV plot for Na_15_Sn_4_/C (80:20) | Na_3_PS_4_ | S (33%)/C (17%)/Na_3_PS_4_ (33:17:50) with an areal loading of 3.6 mAh/cm^2^, measured at two different rates (50 mA/g and 100 mA/g, the latter used for operando XRD), compared with the voltage profile of a conventional liquid-based Na–S battery.^52^

In a departure from this challenge, Tanibata et al. reveal multiplateau discharge profiles in all-solid-state Na–S cells, with the initial formation of amorphous sodium polysulfides (Na_2_S_*x*_) followed by conversion to crystalline Na_2_S.^14^ This finding suggests a complex reaction mechanism with untapped potential for enhancing sodium–sulfur battery performance. The reversibility operation of Na–S cells depend critically on the nature (chemical and mechanical) of the phase transitions between Na_2_S and S.

In this study, we elucidate the complex sulfur conversion mechanism in solid-state Na–S cells with Na_3_PS_4_ (NPS) and Na_4_(B_10_H_10_)­(B_12_H_12_) (NBH) electrolytes using a combined approach of operando scanning microbeam X-ray diffraction, ex-situ soft X-ray absorption spectroscopy and surface-sensitive operando X-ray photoelectron spectroscopy. These techniques enable a detailed examination of the polysulfide evolution, phase transitions, and associated stress dynamics within the cell during operation. We reveal that both crystalline and amorphous polysulfides are formed from solid-state reactions. They include (i) the polysulfides identified in the Na–S phase diagram (Na_2_S_5_, Na_2_S_4_, Na_2_S_2_, and Na_2_S), (ii) high-order polysulfides previously reported in Na–S batteries with liquid electrolytes (Na_2_S_*x*_, where *x* = 6–8), and (iii) Na_2_S_3_, which emerges only at high temperature or high-pressure conditions. These transformations are governed by diffusion-limited kinetics and depend on localized stresses. Beyond elucidating the full Na–S reaction pathway, this work emphasizes the critical role of pressure as a thermodynamic variable in exploring reaction mechanisms while also shaping reaction pathways, which offers new perspectives for optimizing solid-state battery performance.

## 2 Results and Discussions

To reveal the reaction mechanism of sulfur (S) in solid-state Na–S batteries, we performed operando scanning microbeam X-ray diffraction along the cross-section of the cell. This technique not only allows tracking of phases and microstructural changes but also the associated stress evolution in real-time. For performing the operando experiment, solid-state Na–S batteries have been assembled using a blend of Na_15_Sn_4_ (80%) and C (20%) as the anode, NPS as the separator, and a blend of S (33%), C (17%), and NPS (50%) as the composite cathode (areal loading: 3.6 mAh/cm^2^). Details on preparation, assembly, and basic characterization related to materials and cell components are provided in Supporting Information Note 1. Due to time constraints at the synchrotron testing facility, conditions for cell testing need to be chosen to allow cell cycling without losing its electrochemical characteristics. For a current density of 50 mA/g, an initial discharge capacity of 1376.4 mAh/g has been achieved, comparable to previous reports for solid-state Na–S batteries operated at intermediate temperatures.^15^ Interestingly, the voltage profile and differential capacity plot indicate distinct electrochemical signatures that are mostly consistent with those previously reported for conventional Na–S batteries,^16^ already strongly suggesting the prevalence of a multistep reaction mechanism. When 100 mA/g was applied, the discharge capacity dropped to 1249 mAh/g (Supporting Information Figure 2). As a higher current is applied, the drop in capacity comes naturally, along with an increasing overpotential, while the shape of the charge–discharge curves remains similar (Figure 1c). This indicates that a current density of 100 mA/g has no significant impact on the fundamental characteristics of the composite cathode; hence, this condition has been used for the operando experiments.

After identifying the optimal conditions to run the solid-state Na–S battery at the synchrotron, cells were assembled in a self-constructed operando device (Supporting Information Figure 4), which allows experiments to be performed along the cross-section of the whole cell at constant pressure and temperature. The spatial and time evolution of observed phases in the diffraction experiment during charge and discharge, together with the corresponding voltage profile, are shown in Figure 2a. Details about the phase analysis can be found in Supporting Information Note 2. In Figure 2b, the differential capacity (dq/dV) curve is shown in combination with thickness changes in the composite cathode layer and the overall compositional changes. Details about the refinement of diffraction data and their evaluation can be found in the experimental section and Supporting Information Note 2.

**Figure 2:**
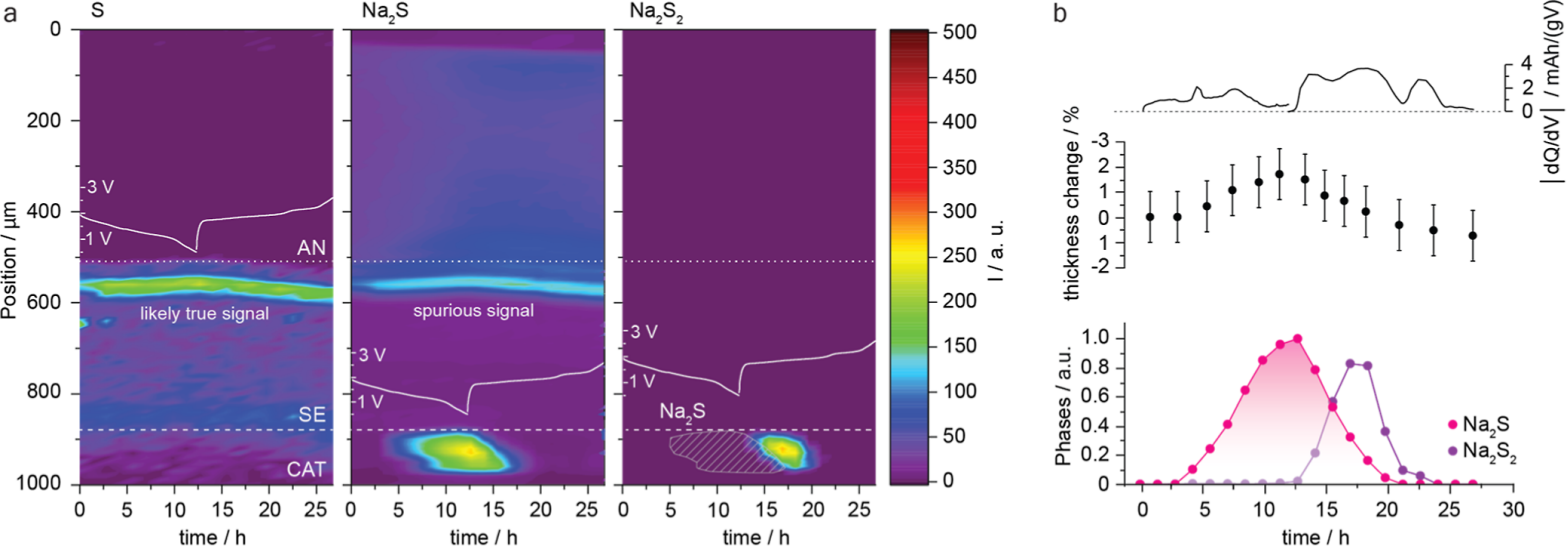
Spatial and time-resolved XRD analysis. (a) Phase maps of the individual compounds: orthorhombic sulfur (S), cubic Na_2_S, and rhombohedral Na_2_S_2_, along with the corresponding voltage profile. The only observable phases are crystalline polysulfides, with sulfur remaining mostly undetectable throughout the experiment. The vertical axis represents the position within the battery cell, in which 0 μm and 100 μm are the position of the current collector. The cathode, electrolyte, and anode region are denoted as CAT, SE, and AN, respectively. The 2D XRD plot for a typical individual scan is shown in Supporting Information Figure 14 (b) Compositional changes across the cross-section over time, correlated with the thickness variation of the composite cathode and synchronized with the dq/dV plot. Not all peaks in the dq/dV plot align with the phases formed during charge and discharge, suggesting the presence of noncrystalline phases.

Due to the preparation by high-energy ball milling, crystalline S transforms into its amorphous phase in the as-synthesized composite cathode.^14^ Initially, a minor amount of crystalline sulfur (Figure 2a) and NPS (Supporting Information Figure 3) can be identified in the diffraction pattern. There is no indication of any phase change that could be associated with the electrochemical signature observed during discharge up to ca. 6 h (1.27 V), while the peak at 7.5 h (1.17 V) can be assigned to the formation of Na_2_S. The absence of signatures for polysulfides, other than Na_2_S, is related to the amorphous nature of long-chain polysulfides (Na_2_S_*x*_; *x* < 6) and their predominately covalent S–S bond character.^17,18^ When further sodiation takes place (Na_2_S_*x*_; *x* = 5,4,2, and 1) the ionic-bond (Na–S) character increasingly favors their crystallization. The formed crystals are constantly growing with increasing degree of sodiation until forming polycrystalline polysulfides. We, therefore, speculate, that polysulfides might already have some degree of crystallinity at lower sodiation levels but remain undetectable due to their small crystallite size. Note, the crystallization process is further highly rate dependent; high discharge rates can also lead to the amorphization of Na_2_S.^19,20^

The reversed reaction peak in the dq/dV plot at about 16 h can be assigned to the desodiation reaction of crystalline Na_2_S (pink line) to crystalline Na_2_S_2_ (purple line). Consequently, we hypothesize that the reverse reaction takes place at about 16 h (1.84 V) due to the symmetrical shape for Na_2_S (assuming similar diffusion-controlled reaction kinetics for both reaction directions). Since the refined quantity of Na_2_S does not match that of the converted Na_2_S_2_ during charge, we assume that Na_2_S_2_ underwent a partial amorphization.

We speculate that this amorphization takes place gradually due to a surface-confined desodiation, resulting from the lower electronic conductivity and larger crystallite size. This surface-confined desodiation causes localized stress accumulation and eventual amorphization. This behavior aligns with observations in lithium–sulfur systems, where limited diffusion and mechanical stress induce structural disorder and phase evolution during cycling.^20^ Since no further crystalline (poly)sulfur species can be observed when further charging takes place, we assume a full amorphization of the polysulfides toward the formation of S_8_.

The first peak observed from 2.1 to 2.0 V in the dq/dV plot might be associated with the reversible decomposition of NPS (Figure 1c; labeled peak (*)). Although it appears reasonable at first glance due to the thermodynamic instability of NPS in the operated voltage range, the phase plots do not show any intensity loss that could support this conclusion (Supporting Information Figure 3). Instead, if this plateau is not related to the electrolyte redox chemistry, the question arises: What does it belong to? We hypothesize that other polysulfides, amorphous in nature, could have been formed as an additional intermediate reaction step. This is reasonable, considering the similar shape of the voltage profile to that of conventional Na–S batteries (Figure 1c).

To identify the presence of further, yet unidentified polysulfides, we performed operando XPS and ex-situ XAS on identical cells, as this approach allowed us to track both crystalline and amorphous compounds. To avoid sulfur species interference from NPS, we used a *closo*-borate electrolyte (NBH) in cell assembly (see experimental section for further details). Replacing NPS with NBH alters the shape of the dq/dV plot (due to the higher Na-ion conductivity of NBH (4.07 mS/cm) compared to NPS (0.59 mS/cm); see Supporting Information Figure 5), though the obvious electrochemical features remain comparable (Figure 4a). Notably, this substitution significantly enhances capacity and cycling performance, even at a high rate of 500 mA/g at 30 °C (Figure 4e). Despite the notable difference around 1.7–1.8 V marked by an additional contribution, closer inspection indicates that the most intense peak at 1.8 V (NBH) could be a superposition of peaks at ∼1.7 and ∼1.8 V. The merging of peaks could be related to the use of the higher Na-ion conductive NBH instead of NPS at given temperatures, indicating a rate-dependency of the conversion reactions. The rate-dependency and associated alterations of electrochemical signatures might also explain the controversial discussion about the reaction mechanism previously.^21^ Moreover, the change of electrolyte also further supports our hypothesis that the minor peak at the beginning of discharge cannot be associated with NPS decomposition.

Operando XPS measurements, shown in Figure 3a, and Supporting Information Figure 6a, reveal that at OCV (2.2 V) the S 2p core level is mainly composed of two relevant components located at 163.6 and 162.1 eV assigned with elemental sulfur (oxidation state S^0^) and reduced sulfur (oxidation state S^1–^).^22^ This slight chemical sodiation of elemental sulfur might be the result of the ball milled cathode composite. During the first discharge, a clear increase in S^1–^ component intensity is observed with decreasing potentials, as shown in the intensity color map in Figure 3a. The integrated area of the different S 2p_3/2_ fitted components allows the calculation of their relative fraction, as shown in Figure 3b. Based on these calculations, the proportion of the S^0^ decreases from 76% at OCV to 61% at 1.9 V, suggesting a stepwise sodiation from Na_2_S_7_ to Na_2_S_5_. At 1.8 V and below, the ratio between S^0^/S^1–^ stabilizes at approximately 50:50, indicating the formation of Na_2_S_4_. Further details on the polysulfide composition calculations are explained in the Supporting Information Note 4.

**Figure 3:**
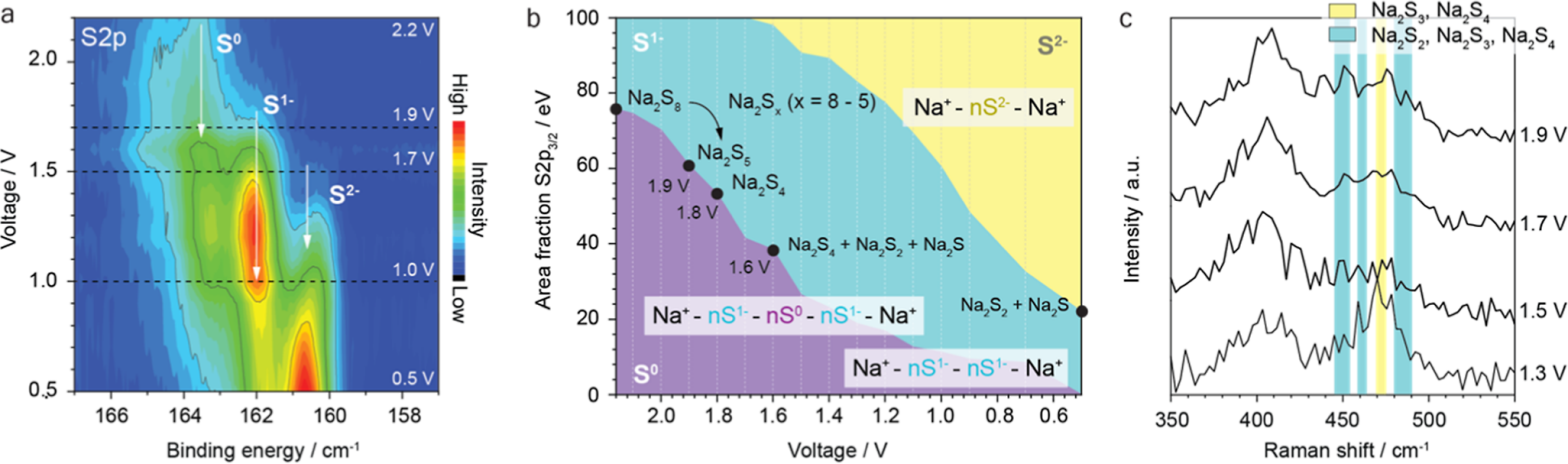
Operando XPS upon first charge. (a) Intensity color-maps of the S 2p core level spectra, showing the evolution of S^0^, S^1–^ and S^2–^ species. All spectra are aligned to the BE position of S^1–^ at 162.1 eV. (b) Area fraction of the fitted S 2p_3/2_ components, calculated for all the spectra during the first discharge. The different polysulfide species and their corresponding onset potentials are highlighted inside the plot. (c) Raman spectra of samples discharged to different characteristic voltages. The highlighted areas correspond to regions where the Raman shift of the polysulfides is expected.^31^

As discharge continues to 1.6 V, the emergence of reduced sulfur S^2–^ attributed to the Na_2_S formation, is observed, while S^0^ progressively diminishes. At 0.5 V, the coexistence of 22% S^1–^ (Na_2_S_2_) and 78% S^2–^ (Na_2_S) is detected. The continuous decrease in the C 1s signal at 284.4 eV (associated with the C–C bonds) during the discharge indicates the coverage of the carbon surface by the polysulfides, as demonstrated in Supporting Information Figure 7a. Simultaneously, the intensity of the polysulfides in the Na 2s core level increases, correlating with the sodiation of sulfur at the surface and the corresponding increase in discharge capacity (see the evolution of the Na 2s core level spectra and the area ratio of Na 2s/Al 2p in the Supporting Information Figure 7b). Additionally, the B 1s core level spectra show no major changes, confirming the electrochemical stability of the Na hydroborate even at 0.5 V. However, a decrease in B 1s intensity suggests that the polysulfides can partially cover the solid electrolyte.

Furthermore, the binding energy (BE) peak shifts of S 2p, Na 2s and B 1s as a function of the applied potential, presented in Supporting Information Figure 7b,c, provide a direct insight into the evolution of surface electrical conductivity for the various formed species.^23,24^ The linear shift of the B 1s confirms the expected electrically insulating nature of the SE. Moreover, both C 1s and S^0^ exhibit negligible shifts above 1 V, confirming their electric conductive character. However, below 1 V both components begin to shift with the applied potential, indicating changes in the surface and interface electrical properties, which become more conductive in conjunction with Na_2_S formation.

Upon charging to 2.8 V, a partially reversible desodiation mechanism is observed. Between 0.5–2.0 V the S^1–^/S^2–^ ratio remains constant at approximately 22:74 on average, with only minor traces of S^0^ starting to evolve at 1.1 V. Only at 2.1 V a sudden jump in the S^0^ appears, coinciding with the disappearance of S^2–^ (Na_2_S) and a stabilized S^0^/S^1–^ ratio of approximately 45:55 (see Supporting Information Figure 6b–d). This suggests the presence of coexisting Na_2_S_4_ and Na_2_S_2_ phases, resulting from an incomplete desodiation at 2.8 V with a relatively low specific charge capacity of 650 mAh/g (see Supporting Information Figure 8). One possible explanation for this incomplete desodiation is the kinetic limitations due to the low applied pressure in the operando cell during cycling (see experiment section of the physicochemical characterization via operando XPS). This insufficient pressure may lead to a partial disconnect and cracking of the composite particles, caused by volume changes during (de)­sodiation. This highlights the importance of maintaining a consistent pressure on the battery stack during electrochemical cycling, to fully observe the reaction mechanism.

Therefore, we apply ex-situ XAS to explore potential reaction steps that remained unidentified during operando XPS, due to the limited utilization of a homogeneous battery stack pressure. Multiple cells were assembled and cycled to characteristic features in the voltage profile, i.e. 0.5, 1.0, 1.1, 1.2, 1.3, 1.5, 1.7, 1.8, and 1.9 V, as well as 2.8, 2.3, 2.2, 2.1, 2.0, 1.9, 1.8, 1.75, 1.7, 1.6, and 1.5 V, under the constant application of 50 MPa stack pressure. The corresponding spectra and analysis are shown in Figure 4b (map) and c,d (quantitative analysis), and Supporting Information Figure 9 (line scan). Details about the spectral fitting and analysis can be found in Supporting Information Notes 3 and Figure 10. The XAS spectra initially resemble those of α-S (elemental sulfur) and a polymeric S_8_ composite. Upon discharge from OCV to 1.9 V, the primary sulfur feature at 2471.5 eV diminishes, while a shoulder at 2469.5 eV, attributed to negatively charged terminal sulfur atoms, becomes apparent. It is a marker for the conversion of S_8_ to long-chain polysulfides labeled as Na_2_S_*x*_ in Figure 4b. The intensity of this shoulder peak at 1.9 V suggests that *x* in Na_2_S_*x*_ exceeds 7, indicating the presence of a mixture of polysulfides with *x* ≥ 6. This agrees with previous reports using in situ TEM on a Na–S nanobattery. They found the formation of amorphous polysulfides with *x* ≥ 6; a clear assignment to either Na_2_S_6_, Na_2_S_7_, or Na_2_S_8_ was, however, not possible.^17^ By continuing discharging to 1.8 V, the intensity analysis of the two main features of sulfur shows *x* ∼ 5, which is in good agreement with previous operando XPS measurements, indicating in Figure 3b at 1.9 V the area fraction between S^0^ and S^1–^ to be 60:40, respectively. Therefore, between 1.9–1.8 V, Na_2_S_5_ is the dominating product. Discharging to 1.7 V marks the existence of Na_2_S_4_. The shoulder peak dominates as the discharge continues, reaching maximum intensity at shorter-chain polysulfides. At 1.5 V cutoff voltage, the analysis shows Na_2_S_3_ as the discharge product. Simultaneously, the primary sulfur feature continues to fade. These observations call to mind those reported for high-temperature solid-state Na–S batteries employing β́́-alumina (300 °C). Similar electrochemical signatures have been obtained and assigned to Na_2_S_5_, Na_2_S_4_, and Na_2_S_3_.^25^

**Figure 4:**
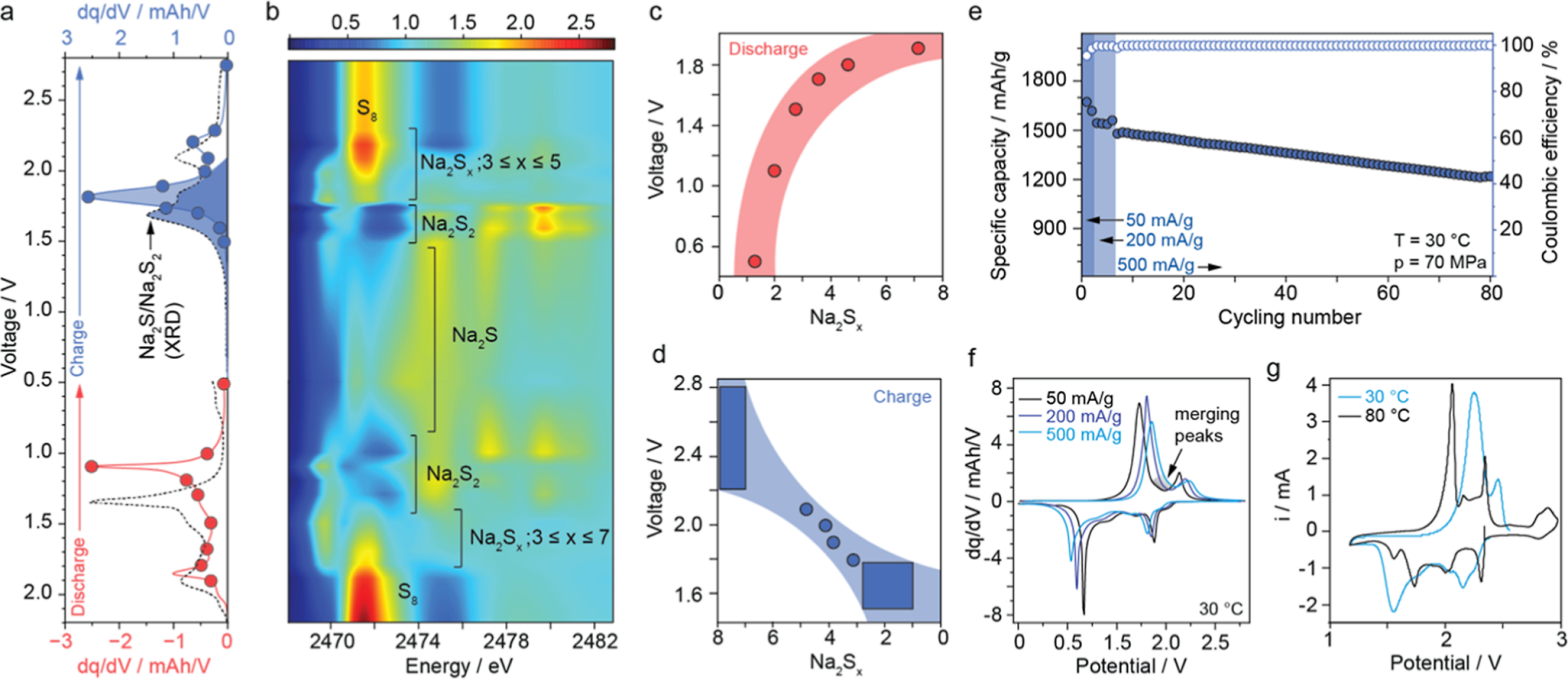
| Ex situ soft XAS and electrochemical analysis (a) dq/dV plot of solid-state Na–S battery employing Na_3_PS_4_ (80 °C; dashed line) or Na_4_(B_10_H_10_)­(B_12_H_12_) (30 °C; solid line). Red and blue dots indicate the voltage where individual cells had been stopped for XAS studies. Blue highlighted peaks indicate the merging of two contributions due to changing kinetics. (b) Contour plot of the ex-situ XAS of the solid-state Na–S battery, showing the discharge cycle and charging cycle. Highlighted regions showing the corresponding XAS spectra of the different polysulfides, grouping the high-ordered Na_2_S_*x*_; *x* = 3–7. (c) Formation plot of the different polysulfides during discharge, and in (d) during charge, showing the formation voltage vs Na_2_S_*x*_ sodiation. (e) Cycling performance of the solid-state Na–S battery employing Na_4_(B_10_H_10_)­(B_12_H_12_) at a rate of 500 mA/g at 30 °C, with a few formation cycles at rates of 50 and 100 mA/g to ensure proper formation of the battery’s internal structure and chemistry. (f) Selected dq/dV plot based on (e) for 50, 200, and 500 mA/g. The arrow indicates peak merging with higher current densities. (g) Cyclic voltammogram of solid-state Na–S battery employing Na_4_(B_10_H_10_)­(B_12_H_12_) at 30 and 80 °C, scanned at 25 μV/s and 10 mV/s, respectively.

Interestingly, this study clearly identified Na_2_S_3_, which is metastable under ambient conditions and decomposes to Na_2_S_2_ and Na_2_S_4_. Compared to Na_2_S_2_ and Na_2_S_4_, Na_2_S_3_ has a low formation enthalpy (4.1 meV/atom) and a stabilizing pressure of 900 MPa, suggesting that Na_2_S_3_ potentially forms when high enough pressure is applied.^26^ This pressure phase sensitivity explains previous success in synthesizing Na_2_S_3_ at temperatures and pressures of about 30 °C and 200 MPa, respectively.^27^ We hypothesize that Na_2_S_3_ can also form in solid-state Na–S batteries when high enough (local) pressure is generated due to the applied stack pressure or significant volume changes associated with the conversion reaction within the composite cathode.

To test this hypothesis, stress evolution associated with polysulfide formation was extracted from diffraction data (Methods; Supporting Information Note 2). Given the amorphous nature of most polysulfides, stress was analyzed using NPS as a kind of internal stress sensor. A complex stress profile emerged within the composite cathode (Figure 5a), driven by conversion reactions and substantial volume changes. These phase transformations and associated volume changes are evident from the regions of diminished and augmented peak intensity maps of Na_2_S, Na_2_S_2_, and NPS (Figure 2a and Supporting Information Figure 3). The composite cathode, i.e., sulfur species, expands during discharge, effectively compressing the separator and anode and vice versa during charge (Supporting Information Note 2 regarding stress analysis). These behaviors are predominantly linked to the Na_2_S_2_-to-Na_2_S conversion process, with other reactions contributing negligibly. Measured stress profiles revealed increasing tensile deviatoric stresses in both the parent NPS and newly formed Na_2_S phases, peaking at 280 and 180 MPa, respectively, near full discharge. This phenomenon may be explained by a predominantly compressive radial stress component and a tensile axial component, consistent with Na_2_S finally precipitating in needle- or leaf-like morphologies within the Na_3_PS_4_ matrix, oriented parallel to the thickness of the cell (Supporting Information Note 2). Indeed, such morphologies have been observed in conventional Li–S systems.^28,29^

**Figure 5:**
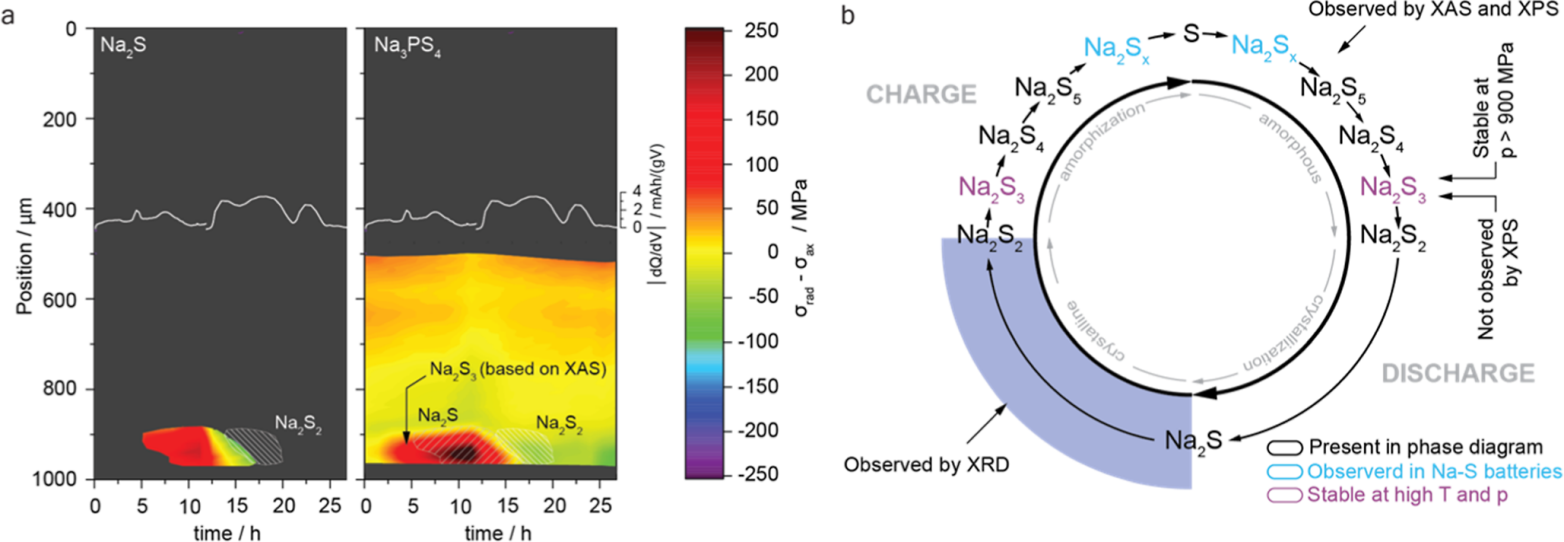
Stress profile analysis derived from diffraction data and proposed reaction mechanism. (a) Time-dependent evolution of stress profiles across the cross-section of the cell during charge and discharge, shown alongside the corresponding differential voltage profile. Stress was calculated using the (100) and (101) diffraction peaks of Na_2_S and Na_3_PS_4_, respectively. Crystalline phases identified in Figure 2a, namely Na_2_S and Na_2_S_2_, are highlighted by shaded areas. Notably, significant stress is generated prior to the formation of crystalline phases, indicating the presence of additional amorphous polysulfides. (b) Summary of reaction pathway during charge and discharge.

Despite the anisotropic stress distribution, the high average axial stress (∼300 MPa) during cell operation supports the proposed stabilization of Na_2_S_3_ under high-pressure conditions. This highlights the critical role of pressure in solid-state batteries on the reaction mechanism, which could significantly impact the cell performance.

Moreover, unlike the recent report proposing that Na_2_S_2_ is thermodynamically unstable (based on the presence of Na_2_S_2_ in ex-situ XAS measurements but its absence in in situ Raman studies),^21^ we observe this phase in both operando XRD and operando XPS (see above), as well as in ex-situ XAS. The observation is again in good agreement with in situ TEM studies of nano Na–S batteries at high and intermediate temperatures, showing that Na_2_S_2_ is a product of the conversion process, even at fast charging conditions.^17^ The absence of characteristic bands in the Raman spectra could arise from methodological limitations of Raman spectroscopy to the cathode composite rather than the nature of the reaction pathway. Raman measurement of samples discharged at different voltages was conducted and is shown in Figure 3c. Most of the polysulfides are amorphous during cycling,^14^ which produces broad, overlapping Raman peaks (440–490 cm^–1^).^30,31^ It is challenging to resolve due to instrument resolution constraints (0.75–4 cm^–1^) and low signal-to-noise ratios exacerbated by dominant carbon/electrolyte signals. Additionally, the strong Raman activity of carbon and laser-induced heating (even at 1–2 mW) risk degrading polysulfides, necessitating ultralow laser power (100–300 μW) that further diminishes polysulfide signal intensity. Between 1.5 and 1.2 V, the main S_8_ feature finally diminishes, while the contribution from short-chain polysulfides further increases. By 1.1 V, Na_2_S_2_ becomes the dominant spectral contributor. At 1.0 V, while some spectral distortion persists, Na_2_S_2_ and Na_2_S appear as primary contributors, potentially alongside longer polysulfides, i.e. Na_2_S_4_ as confirmed by operando XPS. At the end of discharge, Na_2_S is the main discharge product existing with other Na_2_S_*x*_ species as the average x is nearly 1, (though not exactly unity). It is worth noting that the coexistence is expected as the discharge capacity does not reach the theoretical capacity of sulfur. The final conversion step, i.e., Na_2_S_2_ to Na_2_S, provides half of the capacity of the battery but is also associated with the slowest kinetics among polysulfides, making them rate limiting (the slower kinetics also explain the more significant shift of the peak in the dq/dV plot).^32–34^ This observation is also reflected in the S 2p core level spectra obtained during operando XPS measurements from 0.8 to 0.5 V. Here, the BE shift of S^1–^ and S^2–^ reaches their maximum (see Supporting Information Figure 7c), indicating that the overall interface becomes less electrically conductive. At 0.5 V, the S^0^ compound in Figure 3a completely disappeared, leaving only S^1–^ and S^2–^ which clearly confirms the coexistence of Na_2_S_2_ and Na_2_S. Due to the rate-limiting nature of Na_2_S_2_ formation and similar Δ*G* values, it has been suggested that Na_2_S forms directly from Na_2_S_4_ at kinetics-limited conditions.^21^ However, distinct peaks from the dq/dV curves of the discharge process, even at high cycling rates, low temperatures (Figure 4f), and for electrolytes with different ionic conductivities (Figure 4a), can still be observed. Previous studies might have not considered those peaks as significant due to low intensity (note, some peaks have only a 10th of the main peak based on the contributing capacity). Based on our observations and findings for high-temperature Na–S batteries,^17,25^ we conclude that during discharge, sulfur undergoes a sequential conversion to Na_2_S_*x*_, with *x* = 6 to 8, Na_2_S_5_, Na_2_S_4_, Na_2_S_3_, Na_2_S_2_, and Na_2_S.

During charging, from 1.5 to 1.75 V (like 1.3–1.2 V during discharge), the shoulder peak intensifies, with position shifts suggesting low-order polysulfides with a dominant contribution from Na_2_S_2_ (similar to 1.3 to 1.0 V during discharge). As the spectra clarify at higher voltages, more detailed interpretations become possible. At 1.8 V, the spectral profile reflects a mixture of low- and high-order polysulfides similar to 1.5 V during discharge which have been attributed herein to Na_2_S_5_, Na_2_S_4_, and Na_2_S_3_. From 1.8 to 2.3 V, the shoulder feature steadily decreases in intensity, while the S_8_ peak shows continuous growth. Although this feature diminishes, it remains faintly detectable up to 2.8 V. At 2.8 V, with most polysulfides converted to S_8_, the spectra do not fully match the pristine state. This observation mirrors findings in Li–S systems. In the Li system, sluggish solid–solid reaction kinetics and low ionic conductivity limit the full utilization of S_8_, resulting in a final discharge product composed of Li_2_S, Li_2_S_2_, and residual S_8_.^35,36^ During charging, Li_2_S_2_ partially resists reoxidation to S_8_. We observe a similar phase evolution in the sodium system, especially in the cross-sectional phase composition (Figure 2b, bottom), as well as in the operando XPS S 2p core level spectra at 0.5 V (Supporting Information Figure 6b–d) with S^0^ and S^1–^ coexisting. However, we question the previously suggested coexistence of polysulfides^36^ e.g., Na_2_S and Na_2_S_2_ (but also Li_2_S and Li_2_S_2_) thought to result from slow polysulfide redox reactions. This view largely comes from bulk characterization techniques, which may obscure the actual reaction mechanisms by averaging the composition across the entire cathode. We did not observe both phases coexisting at the same location along the cathode cross-section.

Our findings reveal that new phases initiate formation at the separator|cathode interface and progressively propagate toward the current collector. This behavior can be attributed to the evolution of SOC gradients in solid-state batteries, which are linked to the electrical potential gradient across the composite cathode arising from kinetic limitations. This gradient results in an overpotential, which is lowest at the separator|cathode interface, driving the onset of reaction evolution at this location.^37,38^ This suggests that the incomplete utilization of sulfur is not due to limitations in its redox chemistry but rather based on the operational voltage range of the cell, which is insufficiently broad to accommodate the rising overpotential across the composite cathode. The maximum capacity that can be achieved is less than the theoretical capacity of sulfur, even when a constant voltage hold period at the cutoff voltage is applied (see Supporting Information Figure 11). These limitations require potential strategies to improve sulfur utilization, such as optimizing the ionic/electronic conductivity within the cathode composite and engineering graded cathode architectures. The ionic conductivity and Li^+^/Na^+^ transport can be improved by reducing sulfur concentration near the electrolyte interface while electronic conductivity near the current collector side enhances electron transport. The deliberate structural gradient aims to reduce the mentioned overpotential gradients which results in lower polarization and better overall capacity performance at high current densities.

Regarding Na_2_S_4_, it has been suggested that it forms directly from Na_2_S during charging at kinetic-limited conditions.^21^ However, this contrasts with evidence in our work: we observe that changes in kinetics lead to peak merging, as highlighted by the blue peaks in Figure 4a, rather than their disappearance. Further investigation of rate dependency in dq/dV plots, as seen in Figure 4f, shows both increased polarization with higher current densities and merging of peaks. The increasing polarization can be connected to the diffusion limitation in the 2D cathode plane, causing a polarization gradient in vertical space. This kinetic limitation with increased rate causes polysulfide formations to overlap. The kinetic limitation in the Na–S cathode becomes more apparent with a temperature change, shown in the cyclic voltammogram in Figure 4g. At higher temperatures, the formation peaks become more distinct and separated, showing the enhancement toward improved kinetics and diffusion.

## 3 Conclusions

Solid-state Na–S batteries are gaining renewed attention as a promising energy storage technology, offering high energy density, enhanced safety, and cost-effectiveness due to the abundance of sodium and sulfur. Despite their potential, the conversion-reaction pathway of sulfur with sodium in solid-state systems employing an inorganic electrolyte remains poorly understood. In this work, we employ operando scanning microbeam X-ray diffraction, operando X-ray photoelectron spectroscopy, and ex-situ soft X-ray absorption spectroscopy to elucidate the multistep evolution of sodium polysulfides. We uncover the formation of both crystalline and amorphous polysulfides, including species predicted by the Na–S phase diagram (Na_2_S_5_, Na_2_S_4_, Na_2_S_2_, Na_2_S), high-order polysulfides observed in liquid-electrolyte systems (Na_2_S_*x*_, where *x* = 6–8), and phases such as Na_2_S_3_ typically stable only under high-temperature or high-pressure conditions (see the mechanism summarized in Figure 5b). Beyond elucidating the full Na–S reaction pathway, this work also emphasizes the critical role of pressure as a thermodynamic variable in exploring reaction mechanisms and shaping them, offering new perspectives for optimizing solid-state battery performance.

## 4 Experiment Section

### 4.1 Materials Preparation

#### 4.1.1 Synthesis of Na_3_PS_4_ (NPS)

NPS, as the solid electrolyte, was synthesized using a high-energy ball-milling procedure and a subsequent annealing process, as previously reported.^39^ Specifically, Na_2_S (Fisher Scientific 96.18%) and P_2_S_5_ (Sigma-Aldrich 99%) were weighed with stoichiometric molar ratio (3:1) and mechanically mixed in a ZrO_2_ container with ZrO_2_ balls (diameter = 10 mm, ball to powder weight ratio = 28.3:1) in a planetary ball mill (Retsch PM100) at 550 rpm for 3 h. The obtained powder was pelletized (at 150 MPa) and further annealed at 270 °C with a heating rate of 10 °C/min for 2 h in Ar atmosphere.

#### 4.1.2 Synthesis of Na_4_(B_10_H_10_)­(B_12_H_12_) (NBH)

NBH, as the solid electrolyte, was synthesized by high-energy ball-milling and a subsequent annealing process, as reported previously.^40^ Precursors, Na_2_B_10_H_10_ and Na_2_B_12_H_12_, were weight and further mixed in a ZrO_2_ container with an equimolar ratio (1:1) in a planetary ball mill at 500 rpm for 2 h. Then, the mixed powder was pelletized under 100 MPa and annealed at 270 °C under dynamic vacuum for 12 h. The annealed material was pulverized in an agate mortar and further ball milled for 24 h at 660 rpm before using.

#### 4.1.3 Forming Sodium–Tin Alloy

Na and Sn with a stoichiometric molar ratio of 15:4 were added together with 20% carbon (Ketjen Black) in a ball milling jar and further sealed under Argon (Ar) atmosphere. Then, the mechanical alloying was performed for 4 h under500 rpm, as elucidated by Tanibata et al.^41^

#### 4.1.4 Preparation of Cathode Composites

A two-step procedure was conducted to prepare the cathode composite for the all-solid-state Na–S batteries. In the first step, 1 g mixture of Sulfur and carbon (2:1 in weight ratio, respectively) was homogeneously hand-mixed with a weight ratio of 2:1 in an agate mortar for 30 min and further planetarily mixed for 30 min at 2000 rpm. Then, the S/C mixture and NPS (or NBH) were further mixed with a weight ratio of 1:1 for 4 h at 500 rpm to obtain the cathode composite. All of the processes were conducted in an Ar atmosphere. The mass ratio in the obtained cathode composite is 3:1:2 for NPS (or NBH), C, and S, respectively.

### 4.2 Physicochemical Characterizations

#### 4.2.1 Thermal Gravimetric Analysis (TGA)

TGA test was conducted on a STA 449 F3 Jupiter thermal analyzer (Netzsch) within a temperature range of room temperature (21 °C) to 600 °C with the ramping rate of 10 °C/min under Ar atmosphere.

#### 4.2.2 X-ray Diffraction

Samples were loaded into a 10 mm diameter silicon cavity sample support (zero diffraction) and sealed with a dome holder (Bruker) made from polyacrylate to avoid exposure to the ambient environment. The XRD patterns were collected with a 2θ range of 10–70° and a sample rotation speed of 30 rpm on a Bruker D8 Advance goniometer equipped with a Bruker LYNXEYE detector using Cu Kα radiation.

For operando synchrotron X-ray diffraction, the cells have been assembled in a modified custom-made solid-state cell (Supporting Information Figure 4). To begin with, 8 mg of NPS was loaded into the cell cold-pressed at approximately 380 MPa for 3 min. After that, 0.5 mg of cathode composite and 9 mg of alloy anode composite were inserted into different sides of the cells. Finally, the cell was pressed under 150 MPa for 3 min for compacting. A stack pressure of roughly 50 MPa was applied during the charge–discharge process. A controlling system employing a piezo was used to ensure constant stack pressure during cycling. The cross-section image of the battery stack used for this experiment is shown in Supporting Information Figure 12.

#### 4.2.3 Scanning Microbeam X-ray Diffraction

Diffraction experiments were carried out at the high energy materials science beamline (HEMS) side-station P07b operated by Helmholtz Zentrum Hereon at the PETRA III synchrotron of DESY in Hamburg, Germany. A monochromatic X-ray beam with a photon energy of 87.1 keV and lateral dimensions of 500 μm horizontally and 10 μm vertically was scanned repeatedly across the entire stack of cell layers using a scanning step size of 10 μm over a distance of 1 mm, and a resulting scanning period of 0.5 h. This acquisition scheme resulted in an effective thickness-position vs discharge/charge time mapping of the cell. Powder-like diffraction patterns were collected for each mapped point in transmission on a PerkinElmer XRD 1621 Flat Panel area-sensitive X-ray detector with an effective resolution of 2048 × 2048 pixels, placed 1481 mm downstream of the sample. Data were collected within a 2θ range of 0.24° to 9.32°. The exact detector geometry with respect to the gauge volume was calibrated by measuring a NIST LaB_6_ standard powder and employing the routines provided by the pyFAI software package.^42,43^ The mapping of the evolution of phase (trans)­formations was carried out by integrating the intensities of specific visible reflections within a narrow range of diffraction angle 2θ conceptionally equivalent to annular dark-field contrast in a TEM. Additionally, localized background subtraction was performed from within the neighborhood of the specifically investigated reflections, improving the signal/noise ratio for weakly scattering phases. As in the stress evaluation, averaging multiple reflections also served to improve reliability for some phases, applying this approach for 211 and 220 of cubic Na_15_Sn_4_, 110 of cubic Na_3_PS_4_, 111, 220, and 422 of cubic Na_2_S, 200 of rhombohedral Na_2_S_2_ and 113 of orthorhombic S. The resulting peak intensity maps show the presence of a phase (in arbitrary units) with respect to time and the thickness position in the cell. In the case of Na_2_S, this also contains some spurious signals where some reflection of another phase overlaps in a different part of the cell. The ambiguous regions are attributed based on (i) chemo-physical plausibility of encountering a specific phase in a particular region of the layer stack and based on (ii) the possibility of peak overlap or strong amorphous background signal, thus being deemed either ″likely true″ or ″spurious″. Peak intensities plotted in Figure 2a are therefore based on linear-background-subtracted integral peak intensities for the above-mentioned peaks, where minimal overlaps with other phases were present, but could, however, not be excluded completely (See the enclosed Supporting Information Figure 13 showing the theoretical and experimental XRD patterns averaged over the entire experimental scan range and the entire experimental duration).

The measurements of powder samples as reference were performed at beamline BM01 at the Swiss-Norwegian beamlines at the European Synchrotron Radiation Facility. A small amount of sample was loaded into a quartz capillary (Hilgenberg GmbH) and sealed with wax prior to measurement. Beam size of 150 × 300 μm^2^ with a wavelength of 0.78242 Å, and a two-dimensional (2D) detector (2 M Pilatus) were used. The data was integrated by using Bubble software.^44^ The precise detector position was calibrated by measuring a standard silicon reference powder, utilizing the module provided by the pyFAI software package.^43^

#### 4.2.4 Ex-situ Tender-X-ray Absorption Spectroscopy (XAS)

Samples of the sulfur cathode were prepared by discharging and charging the cells to different cutoff voltages under 50 MPa stack pressure. Sulfur K-edge XANES (short energy scans around the S K-edge absorption line) spectra were collected in a transition mode at ASTRA beamline at the SOLARIS National Synchrotron Radiation Center (Krakow, Poland). Athena software (part of the Demeter package) was used to process the raw data (calibration, normalization, background subtraction) and to extract the XANES signal further used for plotting and interpretation (detailed description in Supporting Information Note 3).^45^ A ZnSO_4_ scan was performed between any two separate measurements, and they served as standards for energy calibration. To this end, all the first derivative maxima from the zinc sulfate references were previously aligned to 2480.5 eV to extract the energy shift (correction) to be applied to the sample spectra. No deviation larger than ±1 eV was ever observed. While we measured using both transmission and fluorescence geometries, the former was preferred since we could prepare decent thickness samples that resulted in workable transmission spectra; in this way, we could avoid all the possible interferences linked to the overabsorption in fluorescence, and the quantification would have been more difficult to tackle—hence limiting us to a more qualitative analysis.

#### 4.2.5 Operando X-ray Photoelectron Spectroscopy (XPS)

XPS was carried out during operando mode using the VG ESCALAB 220iXL spectrometer (Thermo Fisher Scientific) with a focused (spot size ∼500 μm^2^) monochromatized Al Kα radiation (1486.6 eV). A comprehensive methodological description of the operando XPS setup, including the design and the assembly of the custom-made operando cell, can be found elsewhere.^23,46^ The operando electrochemical cell accommodated the full battery stack inside an isolating polyoxymethylene (POM) cylindric sleeve (7 mm inner diameter). First 17 mg of the NBH solid electrolyte was pressed at 255 MPa inside the POM. Afterward, 2.2 mg of the cathode composite containing NBH/C/S (3:1:2 mass ratio) was added on the NBH separator and compressed with the Al-mesh as the current collector on top (meshing size of 400 μm^2^) at 150 MPa. Finally, 11.1 mg of the anode composite Na_15_Sn_4_/C (4:1 mass ratio) was added on the opposite side and compressed at 150 MPa. The cell is closed with a lid, allowing good electrical contact while the X-rays can penetrate the cathode composite surface through its open slit, without the disturbance of a current collector. The open slit design of the operando cell results in a nonuniform pressure distribution across the battery stack. Hence no effective pressure control was possible for the operando cell during the electrochemical cycling. During all the operando XPS measurements the WE was grounded to the XPS analyzer.

XPS spectra were conducted at room temperature and under residual gas pressures of ∼2 × 10^–9^ mbar inside the XPS chamber. The spectra were measured at pass energies of 20 eV in the constant energy analyzer mode for S 2p, B 1s, C 1s, Na 2s, and Al 2p core level, without additional charge compensation. All spectra are collected during the potentiostatic cycling of the battery stack at predefined potential steps (see section on electrochemical testing in operando studies). The cycling profile is shown in Supporting Information Figure 8 for the first discharge (2.2 V–0.5 V) and subsequent charge (0.5 V–2.8 V). The data analysis was carried out in the CasaXPS software (Copyright Casa Software Ltd.) and further details on the fitting parameters are provided in Supporting Information Note 4.

#### 4.2.6 Raman Spectroscopy

The ex-situ samples discharged to different voltages (1.9, 1.7, 1.5, and 1.3 V) were opened in the argon glovebox (MBraun) and prepared on a SEM stub for subsequent analysis in the correlative SEM-Raman microscope (Zeiss Sigma 300 with WITec RISE system). To ensure oxygen-free transport from the glovebox, the samples were transferred to the high-resolution SEM in the Leica EM VCT500 transfer system. The Raman microscope used is a WITec RISE, which is fully integrated in the vacuum chamber of the SEM (ZEISS Sigma 300) and therefor all measurements could be done in vacuum. A laser wavelength of 532 nm and a ZEISS LD EC Epiplan-Noefluar 100*x*/0.75­(NA) objective were used. To avoid sample damage by the laser, all samples were cooled to 133.15 K (−140 °C), using a Leica cryo stage and a laser power of only 0.3 mW was used. This low laser power made a long integration time of 180 s per spectrum necessary to achieve a good SNR. To further improve the SNR, spectra from at least three positions on the surface were averaged and all spectra were background corrected using the WiTec 5.2 software.

### 4.3 Electrochemical Characterization

#### 4.3.1 Electrochemical Impedance Spectroscopy (EIS)

The ionic conductivity of the as-synthesized solid electrolyte material was evaluated by EIS. The electrolyte pellet was formed by using a PEEK die mold with stainless steel plungers (TCH Instruments). 80 mg of NPS powder was loaded into the 10 mm diameter PEEK pellet die and pressed at around 380 MPa (3000 kgf) at room temperature for 3 min. Thin carbon layers were introduced between the pellet and the two stainless steel plungers as current collectors for the EIS measurements. Data was collected with a Biologic VMP300 potentiostat, with an excitation potential of 10 mV and a frequency range between 7 MHz and 1 Hz. During the measurement, the sample was put under 50 MPa constant pressure. A special solid-state cell from RHD Instruments was used for the temperature-dependent test. In detail, 115 mg NPS powder was loaded into the 12 mm PEI pellet die and pressed at around 380 MPa at room temperature for 3 min. Two WC plungers were used as a current collector for the EIS measurement. After that, the cell was placed into a CompreDrive instrument for controlling the temperature under 50 MPa constant load. At each temperature, the cell was relaxed for 2 h before EIS measurement. The EIS data was recorded with a Zahner IMex6 potentiostat with an excitation potential of 20 mV and a frequency range between 3 MHz and 1 Hz. The EIS spectra were analyzed by RelaxIS 3 (RHD Instruments).

#### 4.3.2 Galvanostatic Cycling

The cycling tests were performed with a total cathode loading of 5 mg (6.36 mg/cm^2^), corresponding to a sulfur loading of 1.65 mg (2.10 mg/cm^2^). In all batteries, cells were constructed by three layers. 80 mg of as-synthesized NPS (or NBH) (101.86 mg/cm^2^) was first loaded and compressed for 3 min inside the PEEK cell under 380 MPa using a Specac Atlas hydraulic press. After that, 5 mg of the cathode composite was loaded on one side of the pellet, and 100 mg Na_15_Sn_4_–C composite anode was loaded on the other side of the pellet. The three-layer cell was compressed under 150 MPa for 3 min for compacting. The cells were kept in a homemade frame with applied stack pressure of 50 MPa (by screwing the three nuts with 5 N m torque force using a torque wrench) prior to cycling. The applied current for cycling was 164.93 μA (210 μA/cm^2^), corresponding to 100 mA/g. The lower and upper cutoff potentials were 0.5 and 3.0 V. The galvanostatic charge–discharge cycling was performed using a Netware BTS4000 cycler controlled by BTS 8.0 software. All cycling tests were performed inside the Memmert UN30 high-temperature chamber at 80 °C. The capacity of the battery is calculated based on the mass of sulfur. To quantify the specific capacity of the battery cell with a long cycling protocol without contribution from the other components, a cell with a mixture of NBH and C (8:2, in weight ratio) was tested under the same conditions as the cell using the sulfur composite cathode. The cycling performance of this reference cell is shown in Supporting Information Figure 15.

#### 4.3.3 Electrochemical Testing in Operando Studies

##### 4.3.3.1 Galvanostatic charge–discharge

Prior to measurement, the cell was heated up to 80 °C and the temperature was kept constant. During the operando measurement, the cell was cycled using a PalmSens4 potentiostat with a current density of 210 μA/cm^2^ corresponding to 100 mA/g. The data was recorded using PSTrace 9.0 software. A dedicated experimental setup was developed, allowing for operando cross-sectional scanning of model-type battery cells. Potentiostatic charge–discharge*.* The operando XPS cell was connected to a BioLogic SP-200 monochannel unit and cycled in potentiostatic mode, applying predefined potential steps ranging from 100 to 300 mV, while the current was monitored over time. When the current dropped below 1 μA (approximately 2.6 μA/cm^2^), it indicated that the reaction had reached a steady state, at which point XPS measurements were conducted at the corresponding fixed cell potential (see Supporting Information Figure 8).

## Supplementary Material



## Data Availability

The data that support the findings of this study are available from the corresponding author on reasonable request.

## Notes

Operando XRD platform design note, Phase and stress analysis note, XAS analysis note, XPS analysis note, XRD plot of NPS, XRD plot of the anode, TGA plot of the S–C mixture, cross-section image of the densified NPS pellet, Conductivity isotherm plot of NPS, Arrhenius plot of NPS; Cycling performance of battery cells, Spatial and time-resolved XRD plot of NPS and anode, Operando XRD setup, Nyquist plot of NBH and NPS, Operando XPS S 2p core level analysis, XPS plot of elements, Binding energy shift plot, Operando XPS discharge and charge profile, XAS spectra of samples and reference, XAS analysis plot, Discharge curve with a constant voltage hold of the composite cathode, cross-section of the battery stack, XRD pattern of the whole operando XRD battery, 2D XRD plot of a typical scan, cycling performance of the NBH electrolyte (PDF)
