# Supplementary material for: Revealing the Hidden Polysulfides in Solid-State Na–S Batteries: How Pressure and Electrical Transport Control Kinetic Pathways
Source: J Am Chem Soc. 2025 Jun 23;147(27):23492–503. doi: 10.1021/jacs.5c00465 (PMC12257513; doi:10.1021/jacs.5c00465)
Supplement: Supplementary file 1 [file ja5c00465_si_001.pdf]

# Supplementary information

## Revealing the hidden polysulfides in solid-state Na-S batteries: How pressure and electrical transport control kinetic pathways

Hung Quoc Nguyen[1], Mikael Dahl Kanedal[1], Juraj Todt[2], Feng Jin[1], Quyen Do[3], Dora Zalka[4], Alexey Maximenko[4], Dragos Stoian[5], Norbert Schell[6], Wouter van Beek[5], Harald Fitzek[7], Johannes Rattenberger[7], Valerie Siller[8], Steven T. Boles[3], Mario El Kazzi[8], Jozef Keckes[2], Daniel Rettenwander[1,9,10*]

[1] Department of Materials Science and Engineering, NTNU Norwegian University of Science and Technology, 7034 Trondheim, Norway

[2] Chair of Materials Physics, Montanuniversität Leoben and Erich Schmid Institute for Materials Science, Austrian Academy of Sciences, Leoben 8700, Austria

[3] Department of Energy and Process Engineering, Norwegian University of Science and Technology, Trondheim 7491, Norway

[4] National Synchrotron Radiation Centre SOLARIS, Jagiellonian University, Czerwone Maki 98, 30-392, Kraków, Poland

[5] Swiss-Norwegian Beamlines, European Synchrotron Radiation Facility, 71 Ave. des Martyrs, Grenoble 38000, France

[6] Helmholtz-Zentrum Hereon, Max-Planck-Straße 1, 21502 Geesthacht, Germany

[7] Graz Centre for Electron Microscopy (ZFE), Steyrergasse 17, 8010, Graz, Austria

[8] PSI Center for Energy and Environmental Sciences, 5232 Villigen PSI, Switzerland

[9] Christian Doppler Laboratory for Solid State Batteries, Norwegian University of Science and Technology, 7034 Trondheim, Norway

[10] *AIT Austrian Institute of Technology GmbH, Center for Transport Technologies, Battery Technologies, 1210 Vienna, Austria*

[*] Corresponding author: daniel.rettenwander@ntnu.no

**Supplementary Note 1. Platform design.**

XRD has been performed to check the phase purity of the as-synthesized powder. The corresponding XRD pattern is shown in Supplementary Figure 1a. No phase other than NPS can be identified, indicating its phase purity. The as-synthesized powder comprises approximately 1 µm – 3 µm particles and larger agglomerates up to approximately 20 µm (Supplementary Figure 1b,c). When pelletized with a pressure of 380 MPa (the pressure also used for cell assembling), the powder gets densified, whereby certain regions show higher densification than others, and regions of lower densification are interlaced with flaws (Supplementary Figure 1d,e). The heterogeneous densification could be related to the broad particle size distribution.

The Na-ion conductivity of $Na_3PS_4$ (NPS) was investigated by electrochemical impedance spectroscopy within a temperature range of -30 °C to 100 °C. The corresponding impedance data are plotted in a Bode-like fashion in Supplementary Figure 1f. The conductivity isotherms reveal two direct current plateaus, where the high-frequency plateau can only be deconvoluted from the frequency-dependent part with the help of the imaginary part of the modulus. The amplitude of the imaginary part of the modulus is inversely proportional to the capacitance and is, therefore, sensitive to bulk phenomena. Hence, the high-frequency plateau can be assigned to the bulk transport in NPS. The low frequency corresponds then to grain boundaries. The bulk and the total Na-ion conductivity of NPS at room temperature were calculated to be 0.154 mS/cm, similar to the values reported previously.[1] In Supplementary Figure 1g, the temperature dependence of the Na-ion conductivity is shown. It follows Arrhenius behavior according to $\sigma T = \sigma_0 \exp(-E_a/(k_B T))$. The activation energy $E_a$ is 0.21 ± 0.02 eV, similar to previously reported values.[2,3]

To avoid parasitic side reactions between Na metal and NPS due to their thermodynamic instability towards low voltage, a $Na_{15}Sn_4$ alloy anode has been chosen. To improve the electronic conductivity of the $Na_{15}Sn_4$ alloy anode, 20% of carbon has been added. The phase purity of the alloy has been evaluated by XRD. The corresponding XRD pattern is illustrated in Supplementary Figure 1h. All reflections can be assigned to the indented alloy, indicating phase purity.

To ensure uniform distribution, tight contact between sulfur, NPS, and conductive carbon, and a percolating network for electrical transport, the components blend was homogenized by ball milling. The sulfur content in the sulfur-carbon composite is determined by thermogravimetric analysis (TGA). The corresponding curve is shown in Supplementary Figure 1i. The evaporation of sulfur starts at about 200 °C and disappears completely at about

400 °C as indicated by the mass plateau. Hence, the mass fraction can be calculated by the mass loss, which is 67 %, resulting in a sulfur mass fraction in the cathode composite of 33 %.

**Supplementary Note 2. Phase and stress analysis**

A collimated X-ray beam was scanned across the battery cross-section, and Debye-Scherrer diffraction rings originating from NPS were used for phase analysis. The distortion of the Debye-Scherrer rings was employed to calculate X-ray elastic lattice strains. These allow for the evaluation of first-order macroscopic stresses, i.e., stresses that extend over multiple crystallites or even the entire sample. Since the battery is operated under a constant uniaxial stack pressure of ≈ 50 MPa, the axial stress profile along the solid-state Na-S battery (x-direction) can be supposed to be the principal stress component. In such a configuration, the stress difference between axial and radial direction can be evaluated using known X-ray elastic constants and without exact knowledge of unstrained lattice parameters, which is a great methodological advantage. The associated diffraction signal has been selected for stress evaluation since NPS is considered stable against sulfur within the voltage window of interest. NPS has been used in the cathode composite to increase the intensity of the reflections of interest for probing stress. It must be emphasized that the calculated axial-radial stress difference is averaged over the entire probed battery slice (10 μm wide in x-direction, 1 mm in z) since no spatial filters were used to discriminate the diffracted radiation produced at different points of the probed volume.

Starting from the anode material (top), cubic $Na_{15}Sn_4$, the peak intensity maps reveal the thickness decrease of this layer during initial discharging and vice versa during charge. The initial anode thickness is not fully recovered, which could be related to the consumption of Na by parasitic side reactions, consequently causing a lower Na content in the alloy anode. The varying changes in thicknesses are also reflected in the internal stresses (for technical reasons, we can only assess the difference between the axial and the radial stress component, *cf.* Methods section), which grows more compressive due to the expansion of the underlying composite layer during discharge and becomes slightly tensile during the subsequent charging step. The magnitude of measured stress ranges between -26 MPa in close proximity to the current collector at the end of discharge and 28 MPa towards the anode-separator interface at the end of charge. In general, the measured stress differs only by about 1-2 MPa between the top and bottom part of the anode. This difference might be attributed to some friction between the powder and the cell walls introduced by the applied uni-axial pressure during cell fabrication.[4] Incidentally, this friction is also present during the operando measurement,

explaining why the externally applied stress of -50 MPa cannot be seen to the full extent. The chemo-mechanics of the separator (NPS) and composite cathode (NPS, partially crystalline S, amorphous C) are complex, likely attributable to the composite nature of the mixture (see also Supplementary Note 2 regarding stress evolution of the counter electrode). At the start of discharge, there is a pronounced non-linear thickness-dependency of measured stress ranging from 50 MPa from the anode-separator interface to -15 MPa approx. 50 μm away from the current collector at the cathode side. Until the end of discharge, this distribution is shifted towards more compressive values by about 40 MPa, and at the end of the charging half-cycle, the initial state is more or less restored. In the composite cathode (within the bottom 50-80 μm), the complexity is significantly increased due to underlying conversion-type reactions (formation of, e.g., $Na_2S_2$ and $Na_2S$) and associated significant volume changes (Figure 3).

Those changes in volume are evident from the regions of diminished and augmented intensity in the peak intensity maps of NPS, c-$Na_2S$ ($Na_2S$), and rh-$Na_2S_2$ ($Na_2S_2$) (Figure 2 and Supplementary Figure 3). The composite cathode, i.e., sulfur species, expands during discharge, effectively compressing the separator and anode and vice versa during charge. Looking at the measured stress, the situation is difficult to interpret since, apparently, in both the parent NPS and the newly formed $Na_2S$ phases, values are increasingly tensile until peak discharge, reaching 280 MPa and 180 MPa, respectively. A possible explanation is that the radial stress component is vastly more compressive than the axial stress component, resulting in a tensile value for the measured stress. This seems plausible if $Na_2S$ were to precipitate in a needle (1D) or leave (2D)-like fashion within a $Na_3PS_4$ matrix, with axes oriented parallel to the thickness direction of the cell.[5-8]

The growth mechanism, hence the morphology of polysulfides, is governed by the deposition mode, specifically bulk or adatom diffusion, which dictates whether growth occurs in 1D/2D or 3D, respectively.[9] In solid-state Na-S batteries, where the solubility of Na-ions and polysulfides does not play a role with Na-ion concentration in the electrolyte remains constant compared to conventional type of Na-S batteries, adatom diffusion is dominant. The lowest nucleation energy lies perpendicular to the uniaxially applied load, and in combination with high uniaxial pressures, plastic flow, and adatom diffusion are further promoted, favoring the growth of needle-like or planar structures. Consequently, 2D island growth, characterized by faceted sulfides, is predominant. Indeed, such behavior has been observed in other systems.[10,11]

**Supplementary Note 3. XAS analysis**

To reach the depth of discharge (DOD) and the state of charge (SOC) plots quantitatively presented in the manuscript, a series of steps has been carefully implemented and validated. It should be noted that these protocols have been previously described and used in the relevant literature, especially for Li-based systems, including both theoretical calculations and real-life examples (not so many Na cases/examples, on the other hand). The reader is directed elsewhere for more technical details, protocols, and interpretation.[12-17]

A well-defined aspect: the main S feature has been identified and assigned to uncharged neutral S atoms at 2471.5 eV. Moreover, it is well-established that the charge in polysulfides (PS) migrates toward the end of the chain i.e., the two (negatively charged) terminal S-atoms: in consequence, a shoulder peak has been identified and assigned to negatively charged terminal S atoms at 2469.5 eV.

An internal calibration curve has been done using the two relevant $Na_2S_3$ and $Na_2S_4$ (possessing both the shoulder peak and the main absorption feature) standards. The $Na_2S_2$ has not been included in the calibration because it lacks the main $S_8$ peak component. Small shifts of these peaks (in their positions) are accounted for in the calibration. Then, up/down extrapolation to extract values for x in $Na_2S_x$.

As previously described, the ratio of peak areas, not the peak intensities, was used to try to deconvolute the PS using XAS data. We tried to keep a reliable working protocol where a background/edge step function (arctan) was used, on top of which two Gaussians were added as a main ($S_8$-like contribution) and shoulder-peak (PS-like behavior) component—clear physical/chemical meaning.

Finally, we ended up employing a combination of peak-fitting (vide-supra) and linear combination fit (LCF) to cover as many spectra and their corresponding spectral profiles as well as possible (the goodness of fit parameter, R-factor, was always found to be around or smaller than 1-2%). Whenever this analysis was not applicable (e.g., the SOC plot dashed areas), it is mentioned in the manuscript.

DOD dataset: the points from 1.9 V to 1.5 V (both included) could be fitted with the 2-peak approach. The point at 1.1 V converged closely to the $Na_2S_2$ structure, while the last spectrum at 0.5 V has been reconstructed via a linear combination fitting of $Na_2S_2$ and $Na_2S$, and its profile clarifies that there are still some high-order PS remains, and we do not reach 100% pure $Na_2S$ at the end of the DC process. There are a few points, namely 1.3, 1.2, and 1.0 V, where x in $Na_2S_x$ must be inferred by profile interpolation due to difficulties in reconstructing the spectra.

SOC data set: This approach is similar to the one taken for the DOD. Again, we have the portion from 1.8 V to 2.1 V (both included) that converged decently using the 2-peak approach for the fit. The region from 1.5 V to 1.75 V (included) is very similar in nature with the spectra at (1.3, 1.2, and 1.0 V DC) and presents some spectral characteristics that have not been easy to model. On the other hand, the upper voltage region from 2.2 V to 2.8 V (included) seems to show, consistently, an S8-like spectrum, but some reminiscent high-order PS are present even at 2.8 V.

**Supplementary Note 4. XPS analysis**

The fitting of all spectra was conducted using CasaXPS software (Copyright Casa Software Ltd). To deconvolute various compounds, a Shirley-type background subtraction was utilized, and the peaks were fitted using a combination of Gaussian (70%) and Lorentzian (30%) line shapes through the Marquardt fitting method. This process applied a relative sensitivity factor of 1 and a root-mean-square figure of merit. The residual standard deviation (RSD) is approximately 1 for the S2p, B1s, Na2s, and Al2p peaks. The spin-orbit splitting (ΔE) and branching ratio (β) for the $S2p_{3/2+1/2}$ were fixed at $\Delta E_{S2p}$ = 1.2 eV and $\beta_{S2p}$ = 0.5. For the $Al2p_{3/2+1/2}$ of metallic aluminum, the values were set to $\Delta E_{Al2p}$ = 0.44 eV and $\beta_{Al2p}$ = 0.5. Since the compounds detected in the S2p, B1s, Na2s, and C1s core level spectra exhibited compound-specific binding energy (BE) shifts depending on the applied voltage, their voltage-dependent shifts were adjusted according to their BE position at open circuit potential (OCP), with S2p corrected to 162.1 eV ($S^{(-1)}$ position), B1s corrected to 188.1 eV (NBH solid electrolyte), Na2s corrected to 63.6 eV ($Na_2S_x$ (x = 1 to 8) polysulfides) and C1s corrected to 284.4 eV. The detailed core level spectra for S2p are shown in Supplementary Figure 6(a-b) for the first discharge and charge, respectively. Besides the clear signal of $S^{(0)}$ and $S^{(-1)}$ at 163.6 eV and 162.1 eV, respectively, an unidentified compound is present at OCP with $S2p_{3/2}$ at 164.9 eV, which did not further evolve upon sodiation to 0.5 V. This additional compound might stem from a chemical reaction between S and NBH solid electrolyte after ball milling the composite. In Supplementary Figure 7(a-b) the shift-corrected core level spectra for B1s, C1s, Al2p, and Na2s are shown for the first discharge. In addition, in Supplementary Figure 7b the calculated ratio from the area of $Na_2S_x$ (x = 1 to 8) in Na2s and of $Al^{(0)}$ in Al2p is plotted together with the applied working electrode potential ($E_{WE}$) over the discharge capacity of sulfur. The area ratio follows the capacity evolution as a sign of continuous sodiation of sulfur. The evolution of the BE shifts over the applied voltage upon first discharge is shown in Supplementary Figure 7(c-d). The full width at half maximum (FWHM) of the various sulfur components ranged between 1.0 – 1.2 eV for $S^{(0)}$, 1.1 – 1.5 eV for $S^{(-1)}$, and 1.0 – 1.1 eV for $S^{(-2)}$.

Calculations of the polysulfide composition are based on the ratio between the area fraction of $S^{(0)}$, $S^{(-1)}$ and $S^{(-2)}$. The signal of $S^{(-2)}$ can exclusively be assigned to $Na_2S$. Under the assumption that each polysulfide consists of two $S^{(-1)}$ connected to each sodium $Na^+$ ion, we have $n \cdot S^{(0)}$ ($1 \leq n \leq 8$) connecting $S^{(-1)}$ (see the species evolving in Figure 3b of the manuscript). Above 1.8 V, the ratio between $S^{(0)}$:$S^{(-1)}$ follows $Na_2S_8$ (6:2), $Na_2S_7$ (5:2), $Na_2S_6$ (4:2), $Na_2S_5$ (3:2), with their corresponding area fraction of (75:25), (71:29), (67:33) and (60:40), respectively. At 1.8 V, the area fraction is around (50:50) and indicates the predominant presence of $Na_2S_4$ (2:2). Below 1.8 V the area fraction does not follow the trend anymore, which can be explained by the coexistence of multiple short-chain polysulfides, which is why we assume the presence of $Na_2S_4$ ($2 \cdot S^{(-1)}$ and $2 \cdot S^{(0)}$), $Na_2S_2$ ($2 \cdot S^{(-1)}$ and $1 \cdot S^{(0)}$)and $Na_2S$ ($1 \cdot S^{(-2)}$). We could not discern $Na_2S_3$ in our analysis, as in previous experiments it was described as a metastable compound under ambient conditions, difficult to detect experimentally[18], and capable of a spontaneous disproportionation reaction at room temperature to $Na_2S_4$ and $Na_2S_2$, as described by recent first-principles calculations.[19,20] Besides, due to the nature of the operando XPS measurement, no stack pressure was applied during battery cycling. It is therefore not favorable for $Na_2S_3$ in such conditions.[21]

## Supplementary Figures

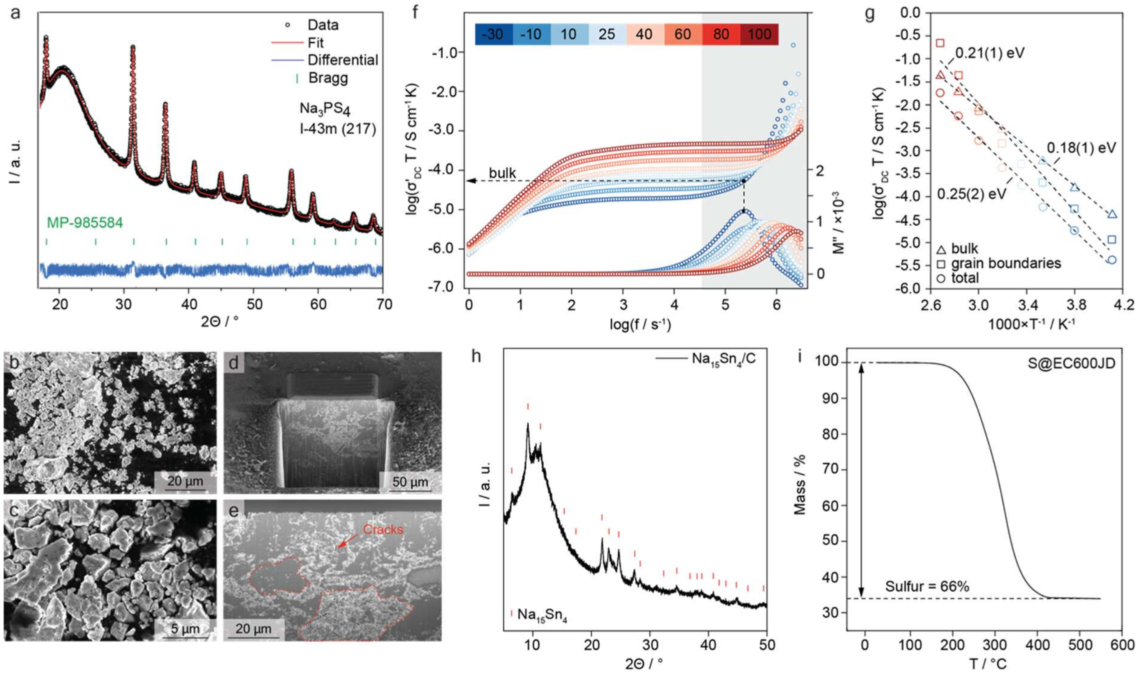


**Supplementary Figure 1 | Design of the platform.** (a) Rietveld refinement of XRD pattern of $Na_3PS_4$ (NPS) powders. The amorphous peak at around 20° is related to the natural characteristics of the dome holder made of polyacrylate. (b-c) SEM images of the NPS powder. (d-e) Cross-section images of the densified NPS pellet. (f) Conductivity isotherms showing the real part of the conductivity σ (left axis) and the imaginary part of the electric modulus M" (right axis) as a function of temperature (-30 °C to 100 °C) are plotted against the frequency. (g) Arrhenius plot with separated bulk and grain boundary conductivity contributions from total conductivity. The data are derived from EIS measurements of densified NPS pellets. (h) XRD pattern of the as-prepared anode. (i) Thermal gravimetric analysis (TGA) of the as-synthesized sulfur-carbon composite shows 66% weight percent of the composite is sulfur.

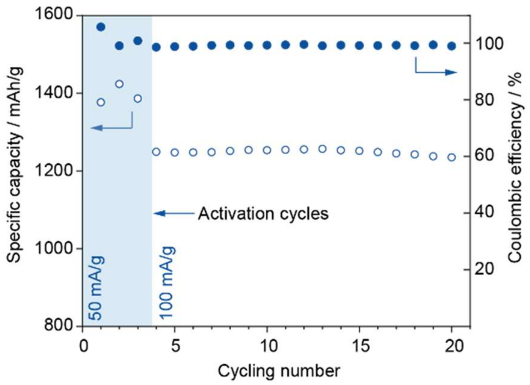


**Supplementary Figure 2 | Electrochemical analysis.** Cycling performance of solid-state Na-S battery employing NPS electrolyte (80 °C) tested in the operando XRD device.

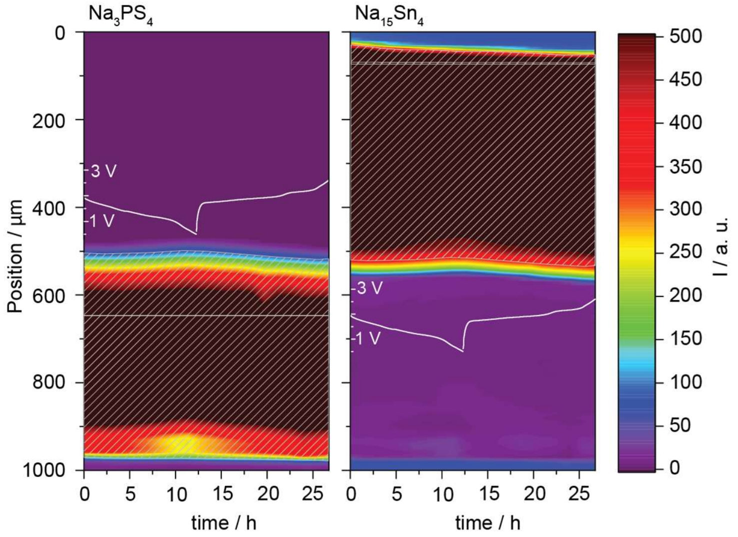


**Supplementary Figure 3 | Spatial and time-resolved XRD analysis.** Phase maps of the individual compounds, identified through qualitative intensity mapping with peak-local background correction: NPS and $Na_{15}Sn_4$, along with the corresponding voltage profile.

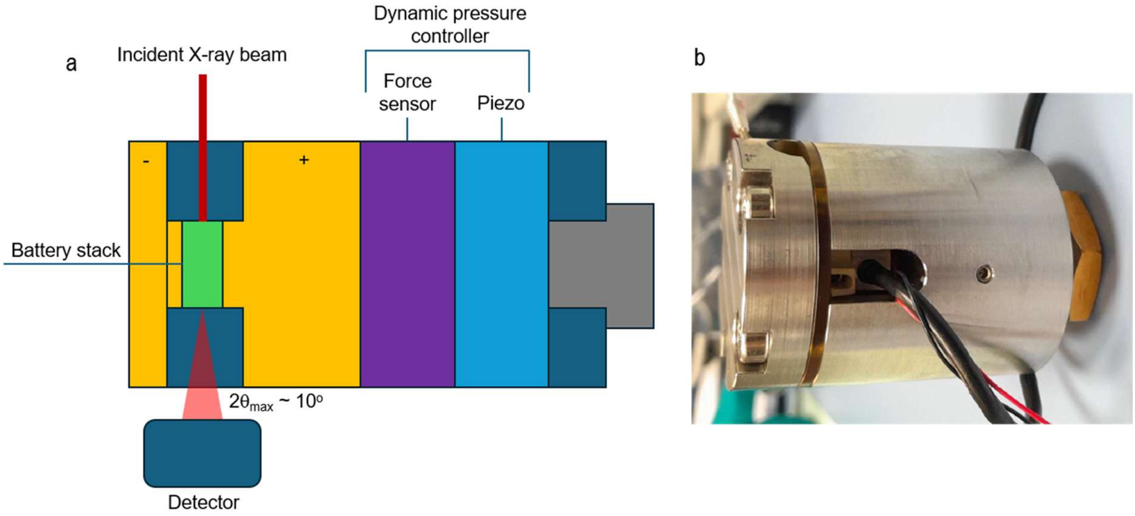


**Supplementary Figure 4 | Operando XRD setup.** (a) Schematic of the setup, which contains dynamic pressure control using a piezo. (b) Photo of the actual device.[22]

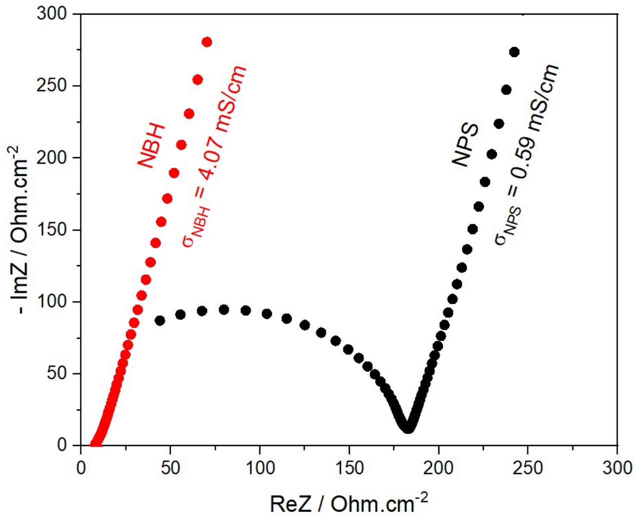


**Supplementary Figure 5 | Nyquist plot of the as-synthesized NBH and NPS.** NBH exhibits around one order of magnitude higher ionic conductivity than NPS (4.07 mS/cm compared to 0.59 mS/cm).

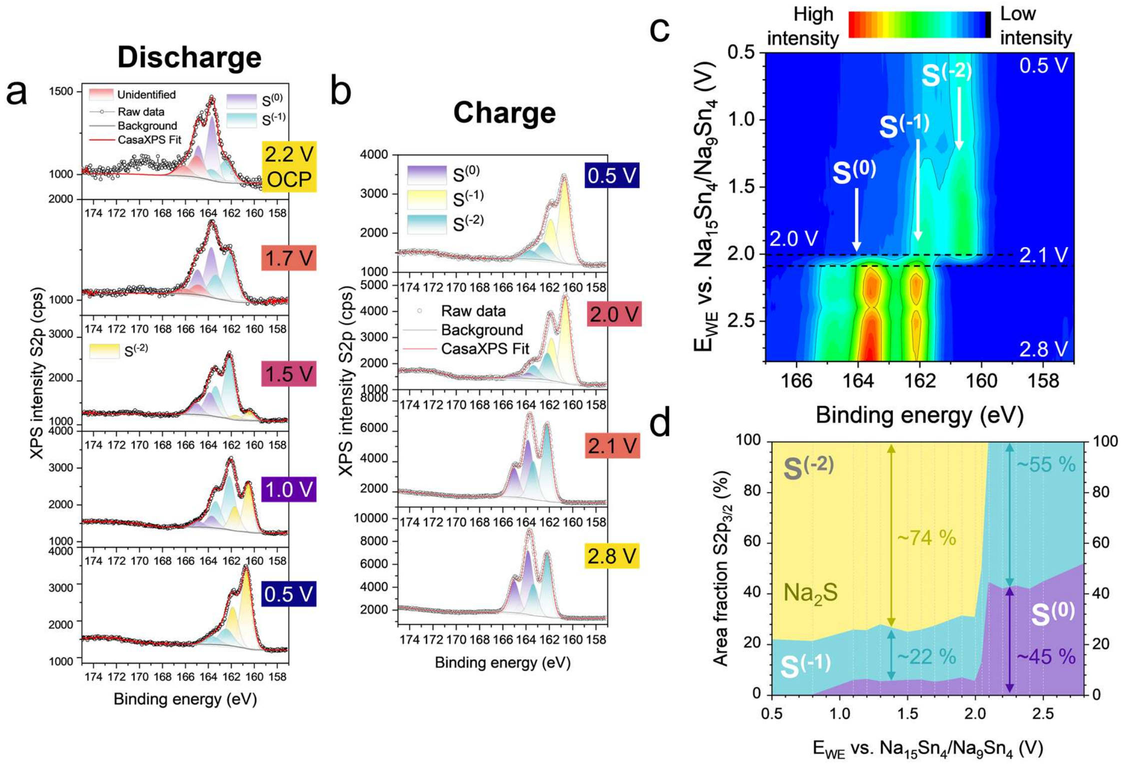


**Supplementary Figure 6 | Operando XPS S2p core level analysis.** (a) S2p core level spectra at different applied potentials upon first discharge with their attributed compounds and corresponding fitting. (b) S2p core level spectra at different applied potentials upon first charge with their attributed compounds and corresponding fitting. (c) Intensity color-maps of the S2p core level spectra upon first charge from 0.5 – 2.8 V. All spectra (a-c) are shift corrected to the BE position of $S^{(-1)}$ at 162.1 eV. (d) Area fraction calculated from the area below the $S2p_{3/2}$ of the fitted compounds for all spectra during the first charge.

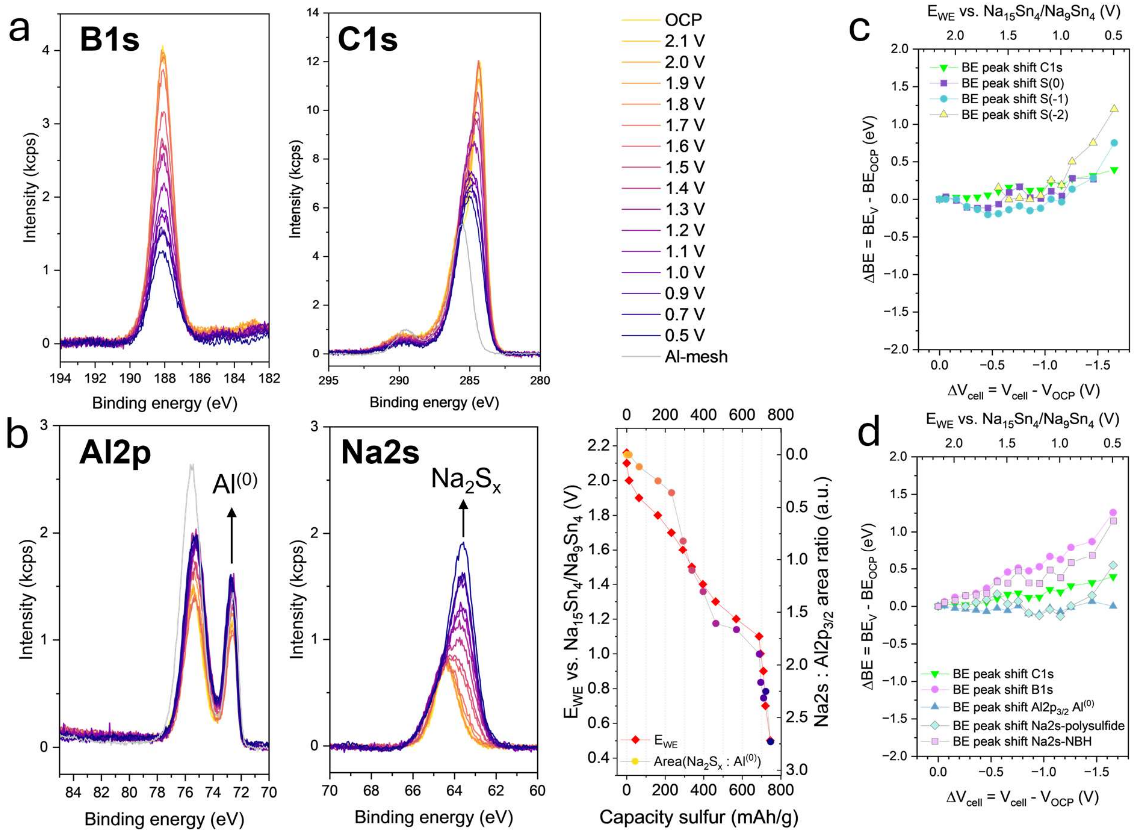


**Supplementary Figure 7 | Operando XPS first discharge.** (a) Evolution of the B1s and C1s core level spectra, and (b) evolution of Al2p and Na2s core level spectra of the cathode composite with applied Al-mesh current collector upon first discharge. All spectra have been corrected for the voltage-dependent binding energy (BE) shifts. In (b) the ratio between the area of $Na_2S_x$ (x = 1 to 8) in Na2s and the area of $Al^{(0)}$ in Al2p is plotted over the capacity of sulfur, together with the applied working electrode potential ($E_{WE}$). (c) The BE shifts extracted from the fitted S2p core level compounds and carbon C-C bonds in C1s over the difference in applied cell voltage. (d) The BE shifts extracted from the carbon C-C bonds in C1s, the B1s of NBH solid electrolyte, the $Al2p_{3/2}$ of the metallic Al-mesh ($Al^{(0)}$), and the Na2s of the polysulfide and the NBH solid electrolyte upon sodiation over different applied cell voltages.

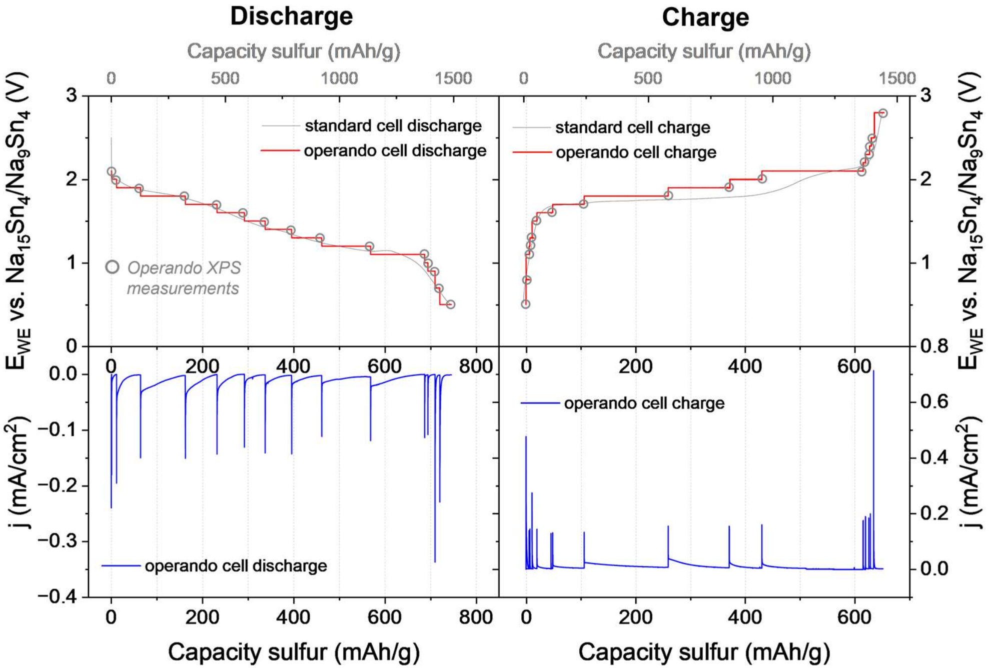


**Supplementary Figure 8 | Operando XPS discharge and charge profile.** The voltage profile for a standard electrochemical cell (grey – galvanostatic) and the operando XPS cell (red – potentiostatic) with its corresponding current response (blue) for each potentiostatic step are plotted over their corresponding capacity normalized to the equivalent weight of sulfur in the cathode composite, for both discharge and charge.

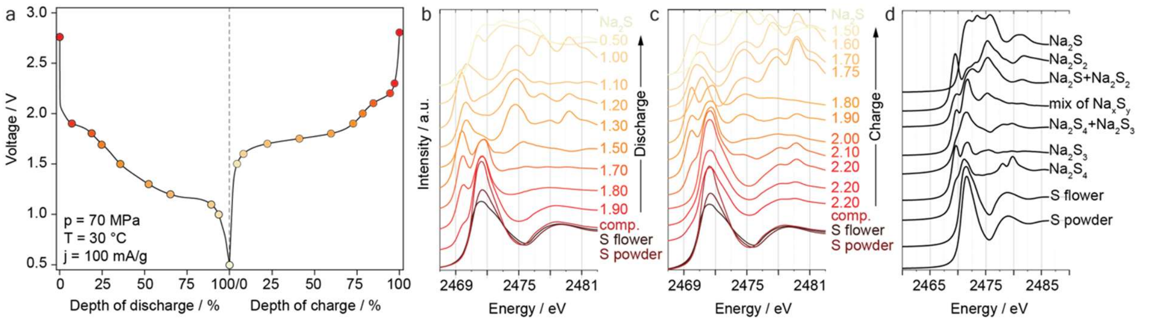


**Supplementary Figure 9 | XAS analysis.** (a) The voltage profile of the solid-state Na-S battery shows the corresponding voltage points from the ex-situ XAS measurements. (b) Ex-situ XAS measurement in discharge and (c) charge, ranging from 1.9V to 0.5V and 2.2V and 1.5V respectively. Showing measurements of pristine composition, sulfur powder, and sulfur flower (mixture of $S_8$ and polymeric S). (d) Simulated XAS spectra of corresponding polysulfide signals. S powder is the commercial sulfur powder, while S flower represents the composite cathode before cycling.

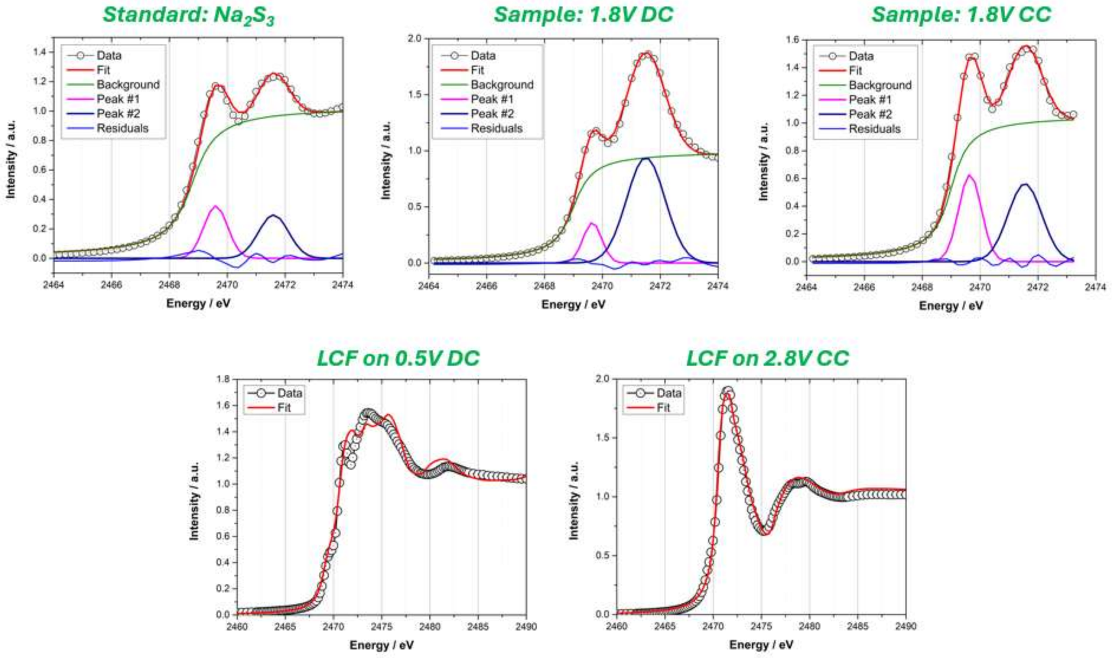


**Supplementary Figure 10 | Exemplification of the working protocols implemented for XAS data analysis on the S K-edge spectra.** A combined approach has been used. It consisted of a peak fit ($S_8$ and PS components) together with a linear combination fitting (LCF) of standards (the ones shown in the manuscript) for cases where the peak fit did not work properly or did not make physical meaning. One example from the standards used for the calibration curve ($Na_2S_3$), one DC sample (1.8 V), and another CC sample (1.8 V) are displayed in the first row. In all the 3 cases, the R-factor indicating the goodness of fit parameter stayed below 1%. The two most relevant examples of LCF employed as a main working horse are presented in the second row. The sample at 0.5 V DC (end of discharge) could be equally well simulated using a mixture of $Na_2S$ and $Na_2S_2$ or a mixture of $Na_2S$ and $Na_2S_2$/$Na_2S_3$. In both cases, ca. 0.73 ratio was assigned to $Na_2S$ while the remaining was either $Na_2S_2$ or half-split between $Na_2S_2$ and $Na_2S_3$ – indicating not a complete conversion towards $Na_2S$ as a sole component (end DC). The R-factor was around 2%. Moreover, the sample at 2.8 V CC (end of charge) presents similar spectra to the S-structure. It could have been well represented (and R-factor below 1%) by a mixture of $S_8$ (ca. 0.83) and amorphous S-component (ca. 0.17). However, minor contributions from some higher order PS (features around 2480 eV) cannot be fully discarded (S purity at the end of the CC cycle).

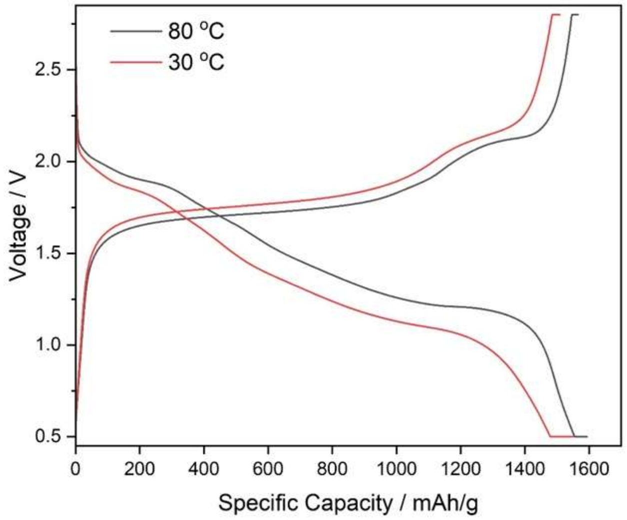


**Supplementary Figure 11 | Discharge curves of the composite cathode under a constant voltage hold at different temperatures.** Two cells were discharged at 30 °C and 80 °C with an applied constant current density of 200 mA/g, followed by a constant-voltage hold at the cut-off voltage until the current decreased to 35 mA/g (approximately 0.02 C, based on a theoretical capacity of 1672 mAh/g). Without voltage hold, the discharge capacities reached 1480 mAh/g at 30 °C and 1556 mAh/g at 80 °C. With voltage hold, these values increased to 1550 mAh/g and 1595 mAh/g, respectively. Despite this improvement, the achieved capacities remain below sulfur's theoretical limit, indicating that the incomplete sulfur utilization is not primarily due to intrinsic redox limitations, but rather constrained by the cell's operational voltage window. Theoretically, elevated temperatures favor the stabilization of $Na_2S_3$, which may partially contribute to the higher capacity observed at 80 °C. However, the charge/discharge curves alone do not allow for a direct correlation with the formation or stabilization of specific polysulfide species. Therefore, the observed capacity enhancement is more likely attributed to reduced internal resistance within the battery components—particularly the electrolyte and electrode interfaces—at higher temperatures.

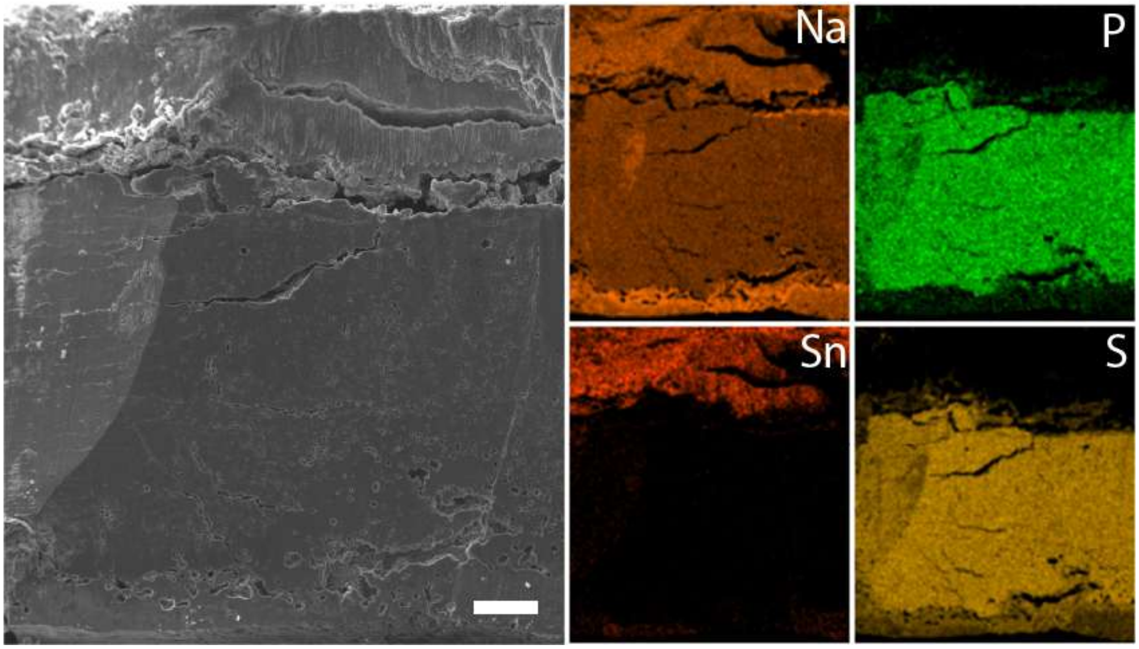


**Supplementary Figure 12 | SEM and EDX characterization of a trilaminar battery stack.** The SEM micrograph reveals three distinct layers, with visible fractures resulting from extraction of the assembly from the operando XRD apparatus. EDX elemental mapping identifies the composition of each layer: the upper region as the anode composite (evidenced by localized Sn signal), the central region as the electrolyte (characterized by prominent P signal), and the lower region as the cathode composite (distinguished by contrast of Na and S signals).

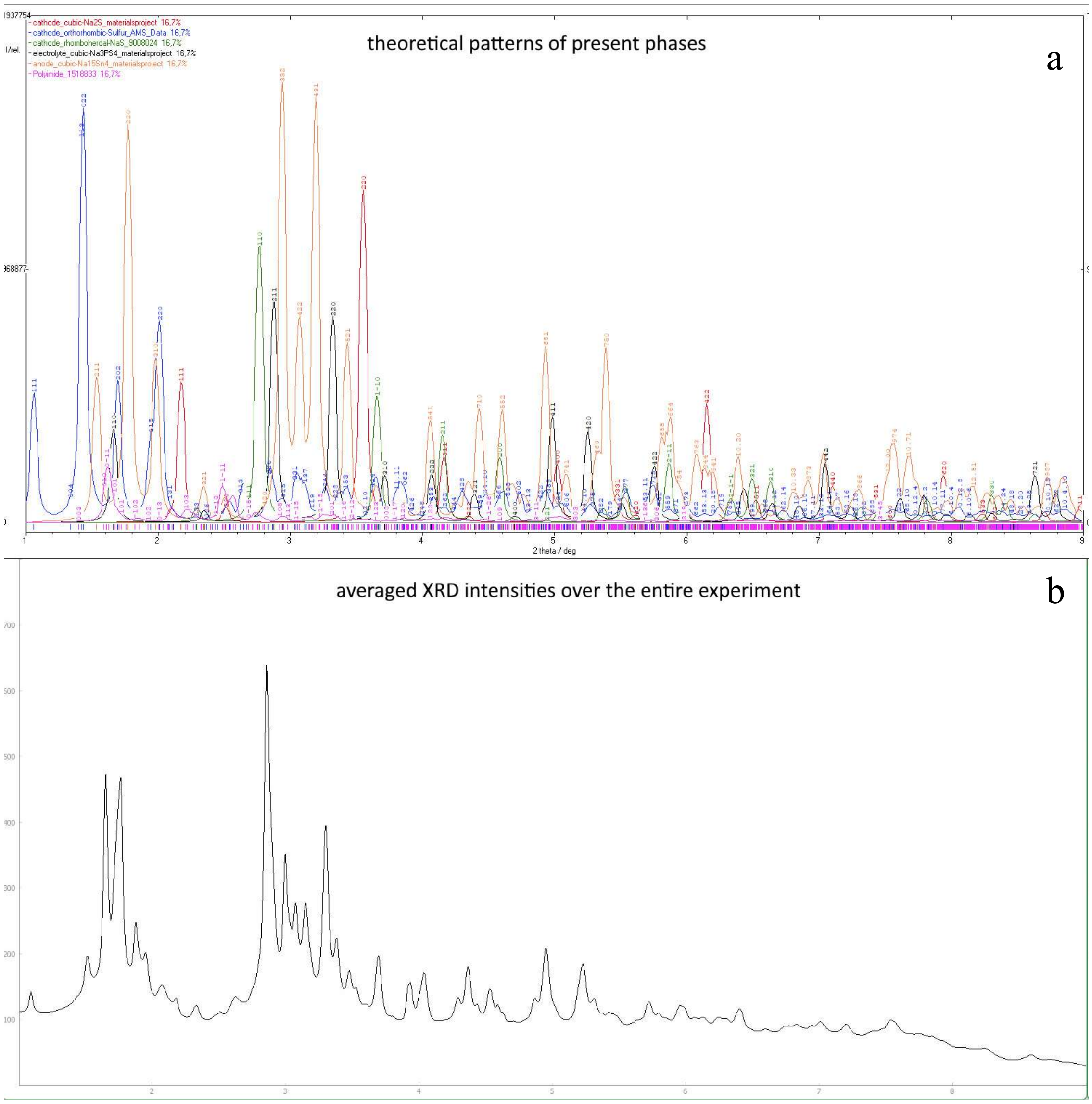


**Supplementary Figure 13 | XRD pattern of the battery stack used for operando XRD experiment.** (a) The theoretical patterns of all present phases. (b) Averaged XRD pattern for the entire experiment (average of all scans and positions).

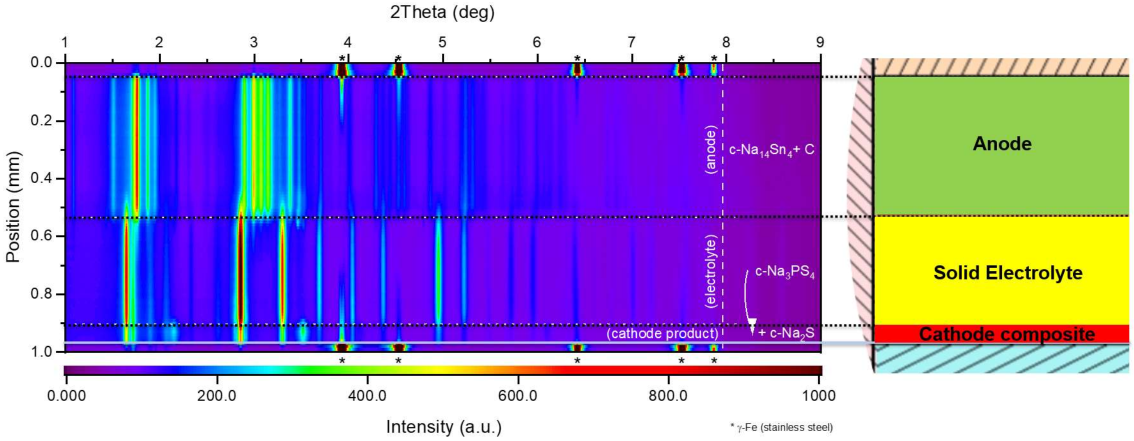


**Supplementary Figure 14 | 2D XRD plot of a typical operando XRD scan throughout the whole cross-section of the battery cell with corresponding cathode, solid electrolyte, and anode region.**

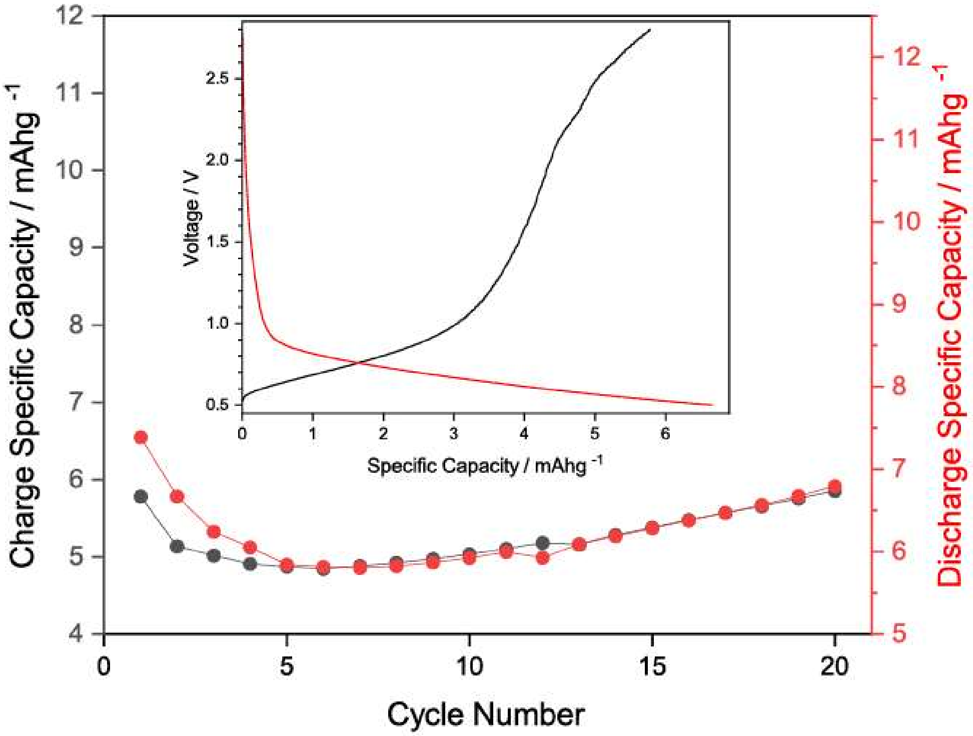


**Supplementary Figure 15 | Cycling performance of a mixture of 80% NBH and 20% C (the inset picture is the charge/discharge curve of the first cycle).** Under the same current density for testing the composite cathode, the mixture of NBH and C achieved only 6.6 mAh/g capacity, which is calculated as 0.66% of the first discharge capacity of the battery in Figure 3e. It further demonstrates that the contribution of NBH to the total reaction of the composite cathode is negligible.

**References for SI**


(1) Hayashi, A.; Noi, K.; Sakuda, A.; Tatsumisago, M. Superionic glass-ceramic electrolytes for room-temperature rechargeable sodium batteries. *Nature communications* **2012**, *3* (1), 856.
(2) Hou, J.; Zhu, T.; Wang, G.; Cheacharoen, R.; Sun, W.; Lei, X.; Yuan, Q.; Sun, D.; Zhao, J. Composite electrolytes and interface designs for progressive solid-state sodium batteries. *Carbon Energy* **2024**, e628.
(3) Yu, C.; Adair, K.; Sun, X. Sulfide-based Electrolytes in Solid State Batteries. **2021**.
(4) Chen, Y. T.; Jang, J.; Oh, J. A. S.; Ham, S. Y.; Yang, H.; Lee, D. J.; Vicencio, M.; Lee, J. B.; Tan, D. H.; Chouchane, M. Enabling Uniform and Accurate Control of Cycling Pressure for All-Solid-State Batteries. *Advanced Energy Materials* **2024**, 2304327.
(5) Ai, G.; Dai, Y.; Mao, W.; Zhao, H.; Fu, Y.; Song, X.; En, Y.; Battaglia, V. S.; Srinivasan, V.; Liu, G. Biomimetic ant-nest electrode structures for high sulfur ratio lithium–sulfur batteries. *Nano letters* **2016**, *16* (9), 5365-5372.
(6) Manthiram, A.; Yu, X. Ambient temperature sodium–sulfur batteries. *small* **2015**, *11* (18), 2108-2114.
(7) Ren, Y.; Zhao, T.; Liu, M.; Tan, P.; Zeng, Y. Modeling of lithium-sulfur batteries incorporating the effect of $Li_2S$ precipitation. *Journal of Power Sources* **2016**, *336*, 115-125.
(8) Zhang, Q.; Yang, T.; Li, Z. Mechanism and Kinetics of Na2Sx (x≤ 2) Precipitation in Sodium-Sulfur and Sodium/(Oxygen)-Sulfur Batteries. *Journal of The Electrochemical Society* **2024**, *171* (1), 010503.
(9) Guo, L.; Oskam, G.; Radisic, A.; Hoffmann, P. M.; Searson, P. C. Island Growth in Electrodeposition. *J. Phys. D: Appl. Phys.* **2011**, *44* (44), 443001.
(10) Li, Z.; Zhou, Y.; Wang, Y.; Lu, Y. C. Solvent-mediated Li2S electrodeposition: a critical manipulator in lithium–sulfur batteries. *Advanced Energy Materials* **2019**, *9* (1), 1802207.
(11) Zhao, C.; Benčan, A.; Bohnen, M.; Zhuo, F.; Ma, X.; Dražić, G.; Müller, R.; Li, S.; Koruza, J.; Rödel, J. Impact of stress-induced precipitate variant selection on anisotropic electrical properties of piezoceramics. *Nature Communications* **2024**, *15* (1), 10327.
(12) Pascal, T. A.; Wujcik, K. H.; Velasco-Velez, J.; Wu, C.; Teran, A. A.; Kapilashrami, M.; Cabana, J.; Guo, J.; Salmeron, M.; Balsara, N. X-ray absorption spectra of dissolved polysulfides in lithium–sulfur batteries from first-principles. *The journal of physical chemistry letters* **2014**, 5 (9), 1547-1551.
(13) Wujcik, K. H.; Wang, D. R.; Pascal, T. A.; Prendergast, D.; Balsara, N. P. In situ X-ray absorption spectroscopy studies of discharge reactions in a thick cathode of a lithium sulfur battery. *Journal of The Electrochemical Society* **2016**, *164* (2), A18.
(14) Wang, D. R.; Shah, D. B.; Maslyn, J. A.; Loo, W. S.; Wujcik, K. H.; Nelson, E. J.; Latimer, M. J.; Feng, J.; Prendergast, D.; Pascal, T. A. Rate constants of electrochemical reactions in a lithium-sulfur cell determined by operando X-ray absorption spectroscopy. *Journal of The Electrochemical Society* **2018**, *165* (14), A3487.
(15) Cuisinier, M.; Cabelguen, P.-E.; Evers, S.; He, G.; Kolbeck, M.; Garsuch, A.; Bolin, T.; Balasubramanian, M.; Nazar, L. F. Sulfur speciation in Li–S batteries determined by operando X-ray absorption spectroscopy. *The Journal of Physical Chemistry Letters* **2013**, *4* (19), 3227-3232.
(16) Gorlin, Y.; Siebel, A.; Piana, M.; Huthwelker, T.; Jha, H.; Monsch, G.; Kraus, F.; Gasteiger, H. A.; Tromp, M. Operando characterization of intermediates produced in a lithium-sulfur battery. *Journal of The Electrochemical Society* **2015**, *162* (7), A1146.
(17) Gorlin, Y.; Patel, M. U.; Freiberg, A.; He, Q.; Piana, M.; Tromp, M.; Gasteiger, H. A. Understanding the charging mechanism of lithium-sulfur batteries using spatially resolved operando X-ray absorption spectroscopy. *Journal of The Electrochemical Society* **2016**, *163* (6), A930.

(18) Oei, D.-G. Sodium-sulfur system. II. Polysulfides of sodium. *Inorganic Chemistry* **1973**, 12 (2), 438-441.
(19) Mali, G.; Patel, M. U.; Mazaj, M.; Dominko, R. Stable crystalline forms of Na polysulfides: Experiment versus ab initio computational prediction. *Chemistry–A European Journal* **2016**, 22 (10), 3355-3360.
(20) Wang, Y.; Hao, Y.; Xu, L.-C.; Yang, Z.; Di, M.-Y.; Liu, R.; Li, X. Insight into the discharge products and mechanism of room-temperature sodium–sulfur batteries: a first-principles study. *The Journal of Physical Chemistry C* **2019**, 123 (7), 3988-3995.
(21) Wan, B.; Xu, S.; Yuan, X.; Tang, H.; Huang, D.; Zhou, W.; Wu, L.; Zhang, J.; Gou, H. Diversities of stoichiometry and electrical conductivity in sodium sulfides. *Journal of Materials Chemistry A* **2019**, 7 (27), 16472-16478.
(22) Nguyen, H. Q.; Todt, J.; Stoian D.; Schell, N.; Redhammer, G. J.; Keckes, J.; van Beek, W.; Rettenwander, D.; to be published.
